# Investigating grandmothers’ cooking: A multidisciplinary approach to foodways on an archaeological dump in Lower Casamance, Senegal

**DOI:** 10.1371/journal.pone.0295794

**Published:** 2024-05-29

**Authors:** Pauline Debels, Léa Drieu, Patricia Chiquet, Jacqueline Studer, Alex Malergue, Louise Martignac, Louis Champion, Aline Garnier, Valentine Fichet, Moustapha Sall, Martine Regert, Anne Mayor

**Affiliations:** 1 ARCAN Laboratory, University of Geneva, Geneva, Switzerland; 2 UMR 8215 Trajectoires, CNRS, France; 3 Université Côte d’Azur, CNRS, CEPAM, Nice, France; 4 Museum of Natural History of Geneva, Geneva, Switzerland; 5 UMR DIADE équipe Dynadiv, Institut de Recherche pour le Développement, France; 6 UMR 8591 LGP, CNRS, France; 7 Department of History, University Cheikh Anta Diop, Dakar, Senegal; 8 Global Studies Institute (GSI), University of Geneva, Geneva, Switzerland; New York State Museum, UNITED STATES

## Abstract

Because they hold information about cultural identity, foodways have been the focus of a variety of disciplines in archaeology. However, each approach documents different stages of culinary preparation and is constrained by the preservation specificities of each type of artefact and ecofact. Difficulties in achieving an interdisciplinary approach may explain the scarcity of such studies. In this paper, we propose a methodology that combines archaeozoological, carpological and microbotanical analysis of ecofacts retrieved in the sediment, with use-alteration, organic residue and microbotanical analysis carried out on pottery vessels, recovered during the excavation of a XX^th^ century archaeological dump site in Lower Casamance (Senegal). The results demonstrate the strength of this multiproxy approach in reconstructing past foodways by characterising the importance of aquatic, terrestrial animals and plant products in the Diola Kassa diet. In addition, this study questions the modalities of food transformation by assessing the preparation techniques of animal and vegetal products (cutting marks, heating processes etc.) and the function of pottery vessels (transport, storage, cooking etc.). Aquatic products and rice were a significant part of the diet of the users of the dump (from archaeozoology, carpology, phytoliths and organic residue analysis) and wet cooking (boiling?), salty and acidic foods seem to have been particularly prevalent (from use-alteration). The absence of specific animal and plant parts in the archaeological record, as well as some pottery function, is also questioned. Beyond gathering the results of each approach, this study focuses on the interweaving of different research methods to depict past foodscape.

## 1. Introduction

### 1.1 The challenge of a multidisciplinary approach of foodways in archaeology

In human societies, food appears as a space of interaction between biological constraints (physiological needs, resources available in the environment) and cultural constructions (representation of what is suitable to eat, transmission of culinary knowledge and know-how, exchanges, etc.), beyond the mere survival of individuals ([1–3]). More than any other physiological function, food practices define the nature of social gatherings ([4] p. 51; [5] p. 421). Because cuisine is practiced daily and highly transmitted, the study of foodways holds information about social identity.

As such, food has often been the focus of archaeological investigations, through various disciplines: archaeozoology, archaeobotany, organic residue analysis, paleopathological studies, ceramology, bone and tooth stable isotopes, etc. Archaeozoology and archaeobotany are the main sources of knowledge about the food consumed by ancient societies, through the animal and plant remains preserved at archaeological sites. In addition, since the Neolithic, ceramic vessels have been a central element in the *chaîne opératoire* of food preparation, storage and consumption. Their investigation through organic residue analysis (ORA) approaches has contributed significantly to the understanding of foodways since they were first implemented in the 1980s (e.g. [6–11]). The morpho-functional study of ceramic objects is likely to document gestures related to cuisine (cooking, mixing, etc.) or the nature of the contents (liquid, acid/fermented, etc.; e.g. [12–15]). Very detailed information is sometimes obtained by microscopic observation of charred residues on the surfaces of ceramic vessels (e.g. [16, 17]).

However, all these approaches do not provide information on the same stages of culinary preparation (choice of plant or animal ingredients, stages of preparation, cooking methods, etc.) and are limited by the preservation specificities of each type of artefact and ecofact. Although researchers have often called for a decompartmentalization, interdisciplinary studies have rarely been carried out. Some studies have confronted the results of two different approaches, such as faunal assemblages and organic residues preserved in the pottery to consider the dietary intake of animal origin (e.g. [10, 18–20]) or botanical remains and organic residues to discuss the plant diet (e.g. [9, 19, 21, 22]), and more rarely three different approaches (ORA and microbotanical remains in pottery and human bone stable isotope [23]). Only a few studies have carried out an in-depth study of the ceramic material by using a combination of morpho-functional analysis, use-alteration analysis and analysis of organic residues absorbed into the walls (e. g. [13, 14, 24, 25]). However, by combining only two or three disciplines, these studies have revealed only part of the food system.

A truly multidisciplinary approach would be required to fully understand archaeological food patterns. The reason for this research gap may lie in the heterogeneity of the data and its degraded and fragmentary nature. Because different conservation criteria, and often mutually exclusive, are required for each approach, crossing investigations is a major challenge. In addition, there are only a few models for interpreting culinary practices from the archaeological record. Ethno-archaeological studies can provide the tools to help interpret archaeological data, by building a reference database based on contemporary societies ([26–28]), but they are rarely coupled with multiple approaches to culinary practice.

### 1.2 A multidisciplinary and integrated approach of food practices in Senegal

The SNF Sinergia project: “Foodways in West Africa: an integrated approach on pots, animals and plants” (PI Anne Mayor, Martine Regert and Tobias Haller) was launched in 2019 with the aim of overcoming this scientific bottleneck. Both archaeological and ethnographic studies have been carried out in Senegal to strengthen methodologies for the study of food practices using solid reference collections. Part of the project was carried out in Lower-Casamance, where food practices are still based on pottery vessels, well documented by previous research ([29]). The field project consisted of two parts: an ethno-archaeological study focusing on pottery and foodways, and an archaeological study that could be linked to the ethnographic data and memory. The archaeological component of the project required the study of a site close in space and time to the ethnographic reference, with good preservation conditions for artefacts and ecofacts, in order to test a methodology capable of revealing past food practices. Dumping areas are considered the most suitable as they offer the opportunity to simultaneously study discarded food remains and associated material culture from a small neighbourhood, reunited in a coherent stratigraphic context ([30]).

An archaeological operation was carried out on the site of “La Poubelle des Mamans” located in the village of Edioungou, in the county of Oussouye (Lower Casamance, Senegal). The site provided a well-documented context for its use through local memory and abundant well-preserved material. It offered a unique opportunity to test a multidisciplinary archaeological approach to food systems, to assess its interpretative potential and to identify its limitations. We present here the results of our integrated study based on archaeological evidence combining archaeozoological, archaeobotanical, ceramological and biomolecular approaches. Archaeozoological, macro- and microbotanical analysis (phytoliths) and organic residue analysis provide direct evidence of consumed taxa and sometimes information on their preparation patterns. The functional analysis of pottery containers (morphometry, use-alteration approach, organic residue analysis and microbotanical study) provides additional data on the nature of cooked food and the modalities of meal preparation. The results of the ethno-archaeological study of pottery and foodways, and their potential for enriching the interpretation of archaeological evidence will be published elsewhere.

## 2. Context, materials and methods

### 2.1. Geographic and cultural contexts

The inhabitants of the village of Edioungou belong to the Diola (or *Jola*, *Joola*, *Jòolas*, *Djola*) ethnic group and Kassa sub-group. Today, people of this group live in Gambia, Senegal (Casamance) and Bissau Guinea.

Some information on Diola foodways is available in the literature. Casamance is historically a rice-growing region ([29] p. 58; [31–34]) and today, rice production accounts for 86% of cereal production, leaving a small share to maize, sorghum and millet ([35]). In addition to its nutritional function, rice has a very important cultural and symbolic dimension; it plays the role of social cement in the group. In terms of meat, fish and aquatic products dominate and any surplus can be processed ([32, 36, 37]). The villagers also breed poultry, pigs, goats and cattle; but these are mainly consumed during festivals and rituals ([29, 38]).

**Fig 1:**
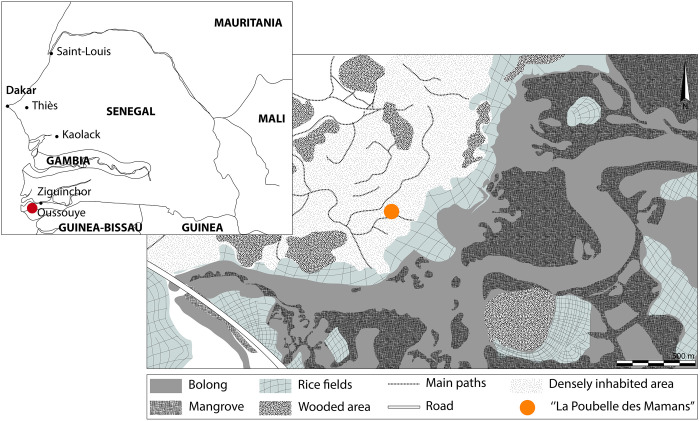
Location of the site inside the village of Edioungou (Lower Casamance, Senegal) and Senegal. Drawing from Google Earth.

The village of Edioungou (Oussouye, Senegal), is located near a small tributary of the Casamance River (Fig 1), in a landscape composed of rice fields and mangroves. The archaeological survey of “La Poubelle des Mamans” was carried out in 2021 in the heart of the village, in an area dedicated to rubbish disposal, shared by neighbouring households. According to the villagers, this dumping ground has been used since the beginning of the XX^th^ century and was abandoned at the very end of the XX^th^ century for sanitary reasons (oral communication, Fabiana Sagna). To confirm this information, ^14^C analysis was carried out on the deepest stratigraphic unit and has confirmed the possible establishment of the dumping area at the beginning of the XX^th^ century, or maybe earlier in the XVIII^th^-XIX^th^ centuries (S1 File; S1 Fig). Recently, it has been partly exploited as a source of material to fill the gullies of the nearby path that form during the rainy season.

### 2.2. Excavation of “la Poubelle des Mamans”

**Fig 2:**
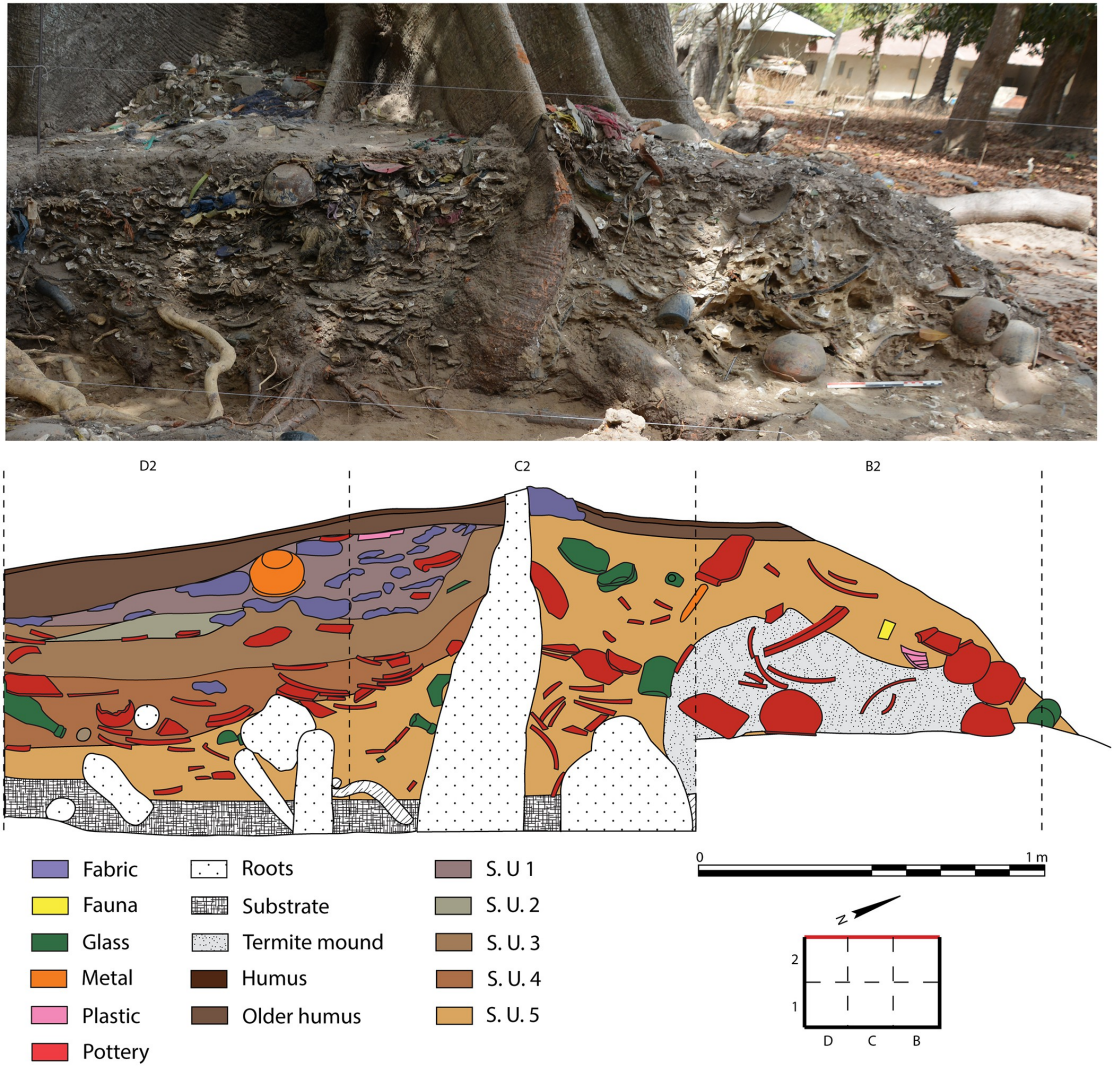
Main section of the survey of "La Poubelle des Mamans". Photograph (up) and drawing (down).

The dumping area extends in a curvilinear shape for about 100 m^2^ between the houses of a small neighbourhood, following the layout of trees planted at the edge of the plots. A 6 m^2^ survey was carried out in the easternmost area which was abandoned at the very end of the XX^th^ century (unlike the rest of the dumping area that is still in use), by agreement with the nearest house. Six arbitrary stratigraphic units were used to divide the survey vertically. The study of the stratigraphic section revealed the presence of an initial heap that gradually built up around the large roots of a kapok tree (*Ceiba pentandra (L) Gaertn*.), and then a more recent addition, probably following a hiatus, covering the entire area. While the first heap yielded oyster shells, glass and ceramic sherds, metal elements, faunal and carpological remains and, depending on the depth, plastic elements, the latter consisted mainly of discarded clothing and plastics (Fig 2).

Vessel fragments (over 5 cm), mostly pottery sherds, and animal bones (over 2 cm) were mapped and collected during the excavation. The ceramic assemblage contained a total of 468 reconstructible pots, but only 161 were sufficiently well preserved to provide full morphometric data. Metal, glass, enamel and plastic artefacts were photo documented, but they are not included in the present study. A total of 1010 bone remains representing over 3 kg of material were collected during the excavation (hand-collected material, S2 File; S2.1 in S2 Fig). Some stratigraphic units (S. U. 3 and 4) consist almost entirely of oyster shell fragments with very little sedimentary matrix, while other units (S. U. 1) yielded almost no malacofauna. Due to their very high abundance and their highly fragmented state, oyster shells were documented stratigraphic description but not collected.

During the excavation, 8 soil samples (12 litres each, 96 litres in total) were collected. They were floated and sieved to collect small faunal remains, charred seeds and possible charred food residues. Over 1200 bone remains and a large number of shell fragments were found. In addition, 37 archaeobotanical items and 184 fragments of carbonised amorphous residue were recovered.

### 2.3. Investigation method

**Fig 3:**
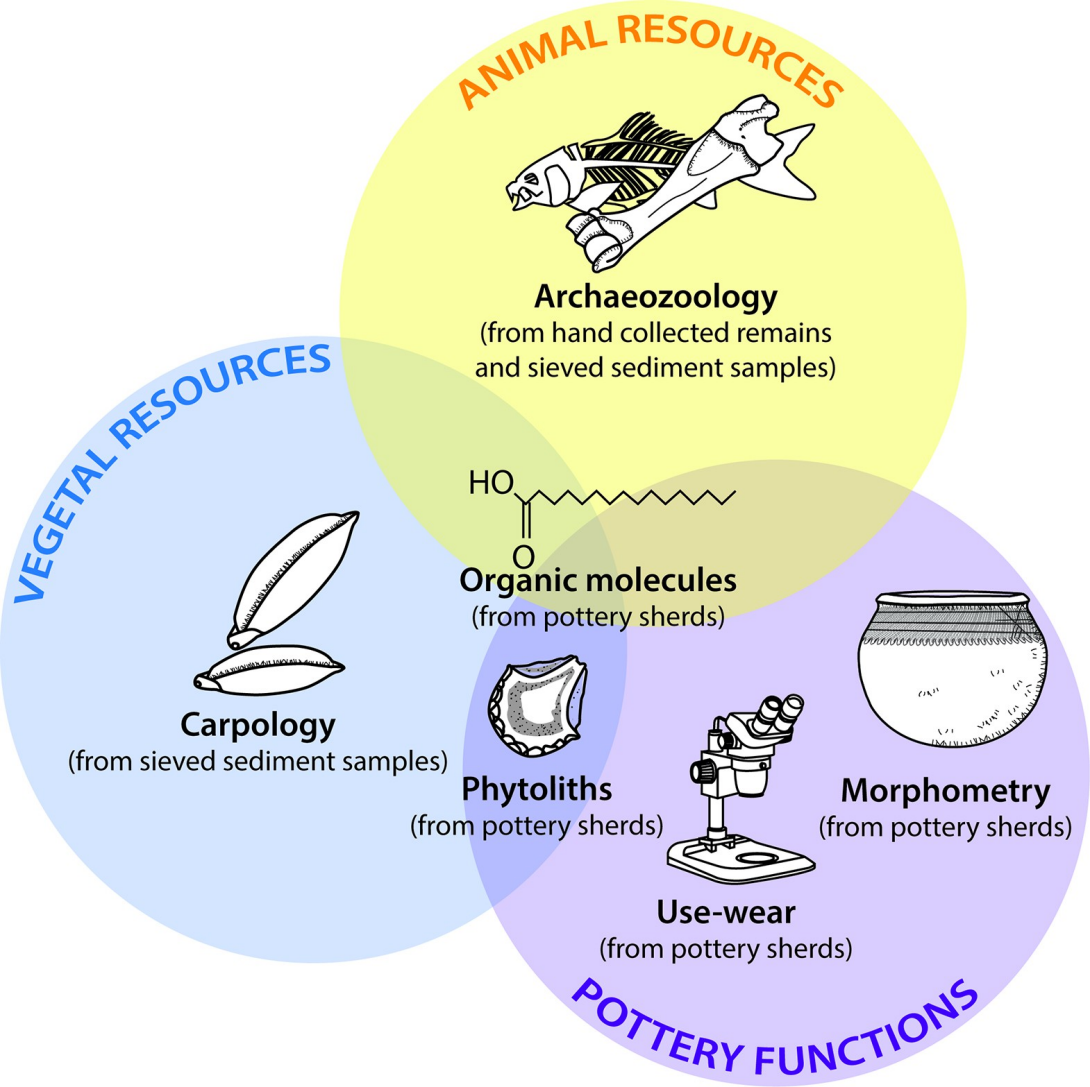
Methods used to identify food resources and their preparation at the Poubelle des Mamans dump site.

Animal food resources were studied using an approach that combined archaeozoological studies and the analysis of organic residues absorbed into the walls of 16 pottery vessels (Fig 3). The latter were also used to investigate the plant component of the diet, in combination with carpological analyses of seeds from the sediments and a study of the microbotanical remains (phytoliths) preserved in the walls of 15 ceramic pots. Food preparation methods were investigated on the pottery by combining morphometric and use-alteration studies with the results of organic residue analyses and identification of microbotanical remains.

**Fig 4:**
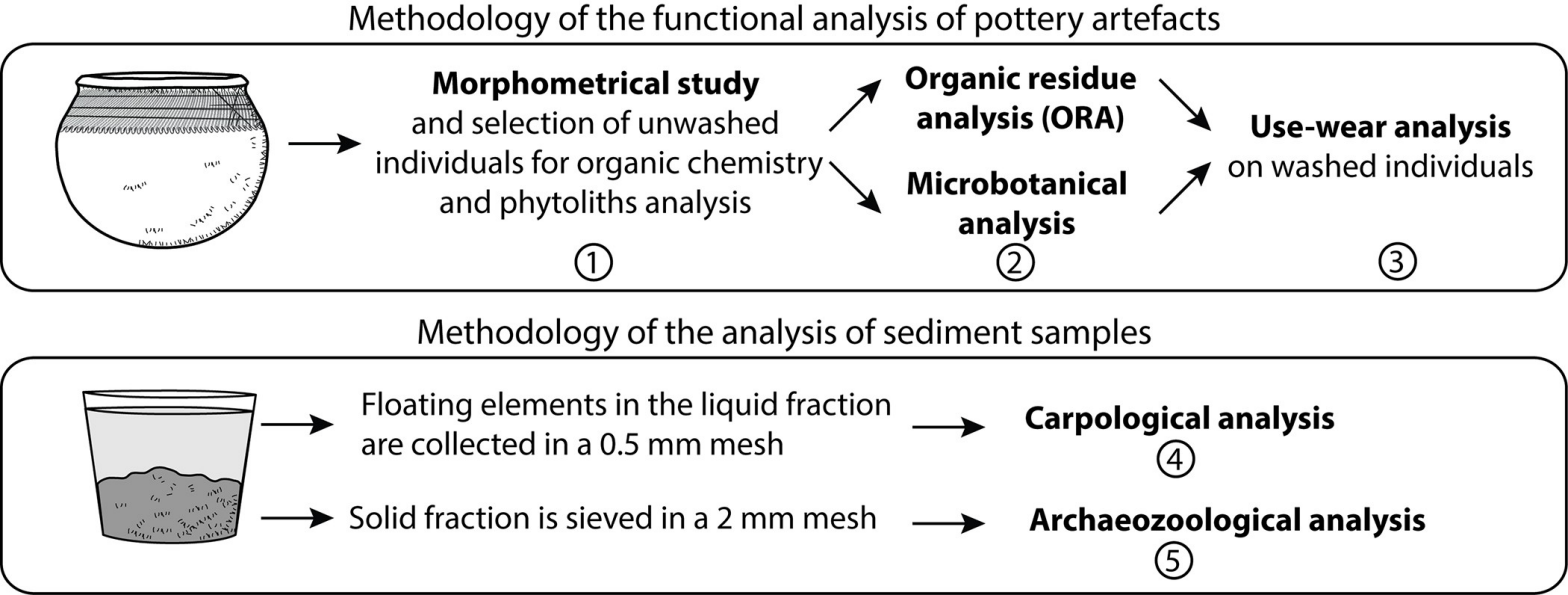
Methodology for a multidisciplinary analysis of pottery sherds and sediments.

As the sediment was the focus of two different investigations (archaeozoology and carpology) and the ceramic assemblage of four complementary approaches (morphometry, use-alteration, microbotanical remains and organic residue analysis), the planning of an effective timeline for each was essential (Fig 4). Sediment samples were soaked and mixed by hand in order to bring charred elements to the surface of the water (Fig 4). The liquid fraction, containing charred remains, was poured into a 0.5 mm mesh sieve while the solid fraction, containing bone fragments, was sorted manually under a stereomicroscope.

**Fig 5:**
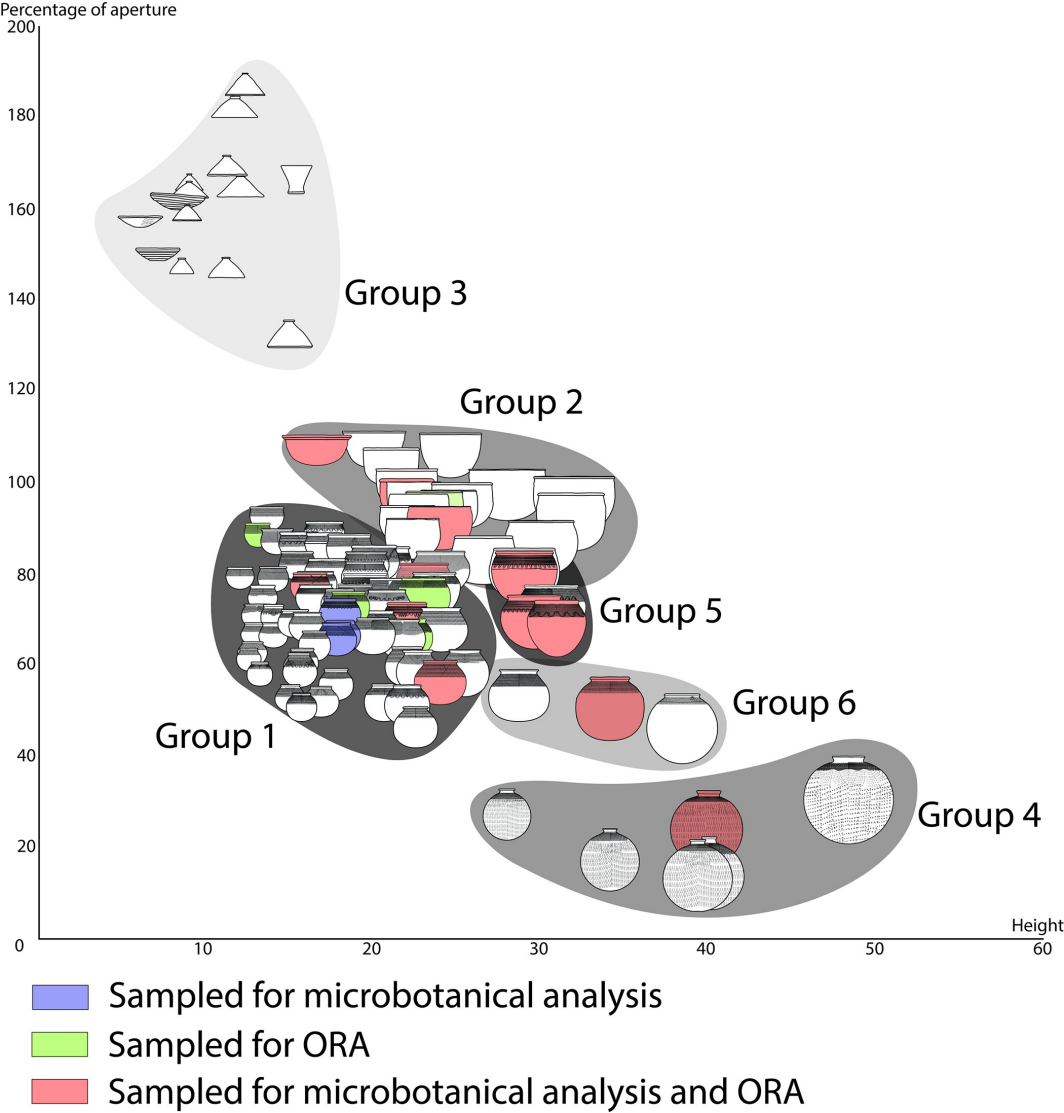
Selection of pots for the analysis of microbotanical remains and organic residues analysis (ORA) among the morphological range at the Poubelle des Mamans.

Regarding pottery, a total of 468 unwashed distinct reconstructible individual vessels were studied from a morphometric point of view. Of these, a total of 161 showing the most relevant morphometric criteria (opening minimum inner diameter, maximum diameter and height) were sampled. The study of these potsherds allowed the creation of six morphometric groups (S4 File), which incidentally correlated with the decoration techniques. These groups were then studied from a use-wear perspective to assess morpho-functional groups. They helped to guide the sampling for organic residue and phytolith analysis (Fig 5). Secondly, preliminary trends in use-alterations in each group led to a question-driven sampling of the unwashed sherds based on the different morpho-stylistic groups. Thirdly, some pots were investigated using organic residue (wall of 16 pots) and microbotanical analysis (wall of 15 pots) (Fig 5), while the remaining sherds were washed and subjected to a second, more thorough, use-alteration analysis.

The specific methods and results of the different disciplinary investigations aforementioned are described in detail in the supplementary informations (S2–S6 Files).

## 3. Results and discussion

### 3.1. Animal resources

#### • The contribution of aquatic animal products

**Fig 6:**
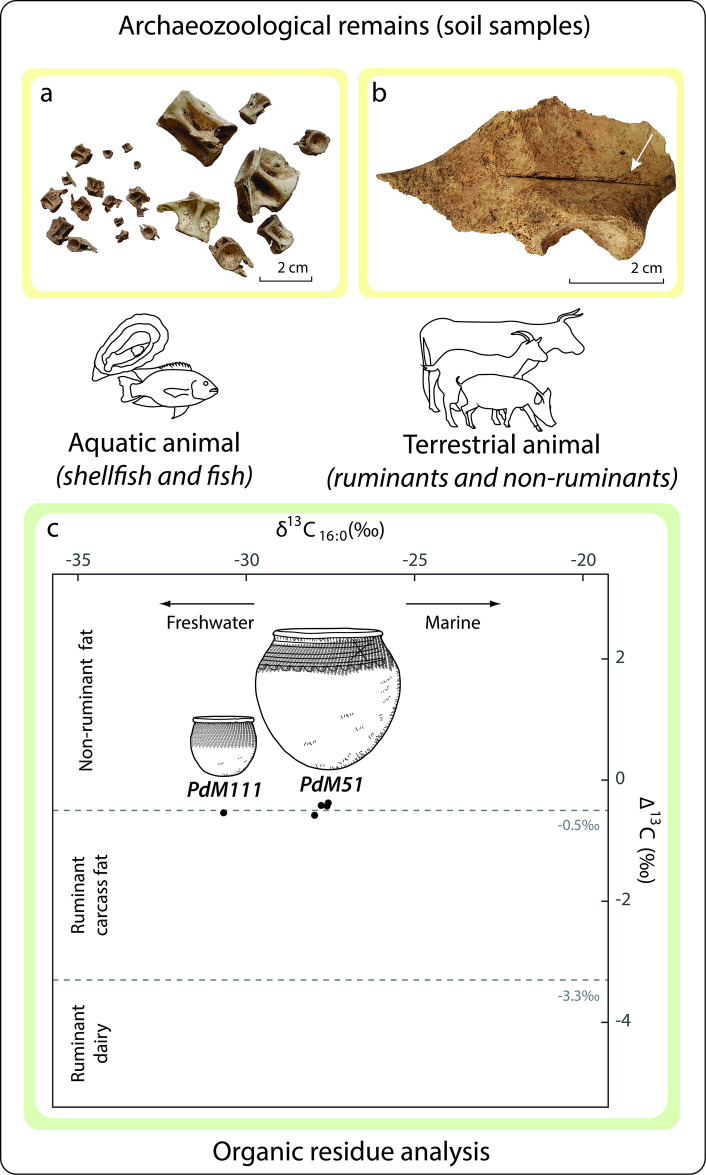
Evidence of animal consumption at the Poubelle des Mamans site: a) Fish bone from the sieved sediment samples (left) and hand-collected (right); b) Pig coxal bone with percussion mark (indicated by an arrow); c) Δ^13^C (δ^13^C_18:0_-δ^13^C_16:0_) values of lipid extracts plotted against their δ^13^C_16:0_ value.

Vertebrate remains are largely dominated by fish, both in the hand-collected ecofacts (S2.1 in S2 Fig) and in the sieved samples (S2 File). In the former, they represent well over half of the faunal assemblage (ca. 59% of total remains and 74% of identified remains, Fig 6). The fishes come mainly from individuals with a total length of more than 30 cm. The largest fish identified is a 70 cm long *Polydactylus quadrifilis* (Cuvier, 1829), known as Giant African threadfin, a species from the Eastern Atlantic. Common in the *bolongs* (river tributaries), a few catfish are identified (some over 50 cm long) as well as smaller cichlids and mugilids. The sieved samples increase the representativeness of the study by further demonstrating the importance of fish consumption, especially those less than 10 cm in length (S2 File). Of note, although the numerous oyster shells could not be collected or counted during the excavation, they must be remembered in order not to underestimate their consumption as part of the Diola foodways.

ORA also suggests the presence of freshwater product in one vessel (PdM 111), where palmitic and stearic fatty acids are highly depleted in ^13^C (-30.4‰ and -30.6‰ respectively; Fig 6 and S5.3 in S5 Fig) ([39]). Some compounds are consistent with aquatic or ruminant products (odd-numbered branched fatty acids C_15:0_br and C_17:0_br, unsaturated fatty acid C_17:1_, phytanic acid with 72% SSR isomer; S1 Table) and the large amount of C_16:0_ and C_18:1_ is consistent with aquatic or plant products (S5 File, S1 Table, S5.2 in S5 Fig) ([39–42]). As not all aquatic markers are identified (pristanic acid, 4,8,12-trimethyltridecanoic acid (TMTD), ω-(o-alkylphenyl) alkanoic acids, markers of thermal transformation of aquatic products), we cannot rule out the possibility that the vessel actually contained ruminant fat and plant products (S5 File).

#### • The contribution of terrestrial animals

Mammals represent only 20% of the identified hand-collected faunal remains but represent 89% of their weight (S2 File, S2.1 in S2 Fig). Pig is the most important in terms of the number of remains, while cattle and carnivores are the next most common taxa, but present in much smaller quantities compared to pig (S2.1 in S2 Fig). Regarding the weight of the remains, cattle make up for a larger proportion than pigs. Goats remains are rare, with only one bone identified. Remains of wild species, such as antelopes, hares and reptiles, are occasionally represented (about 4% of the total assemblage). Birds (chicken or guinea fowl) are also attested but are a very minor component of the assemblage (3% of the identified remains).

In four pottery vessels (PdM 3, PdM 49, PdM 51 and PdM 78), the large amount of lipids (118.0 to 240.2 μg g^-1^, after DCM/MeOH extraction, S1 Table) suggest the consumption of animal fats (Fig 6, S5 File and S1 Table). Single-compound stable carbon isotopes show very similar values for the four samples (δ^13^C_16:0_ around -27‰ and δ^13^C_18:0_ around -28‰), suggesting a mixture of different fats, possibly ruminant carcass fats, porcine fats and/or aquatic products (Fig 6 and S5.3 in S5 Fig). Branched odd-numbered fatty acids and phytanic acid (65–69% SSR isomer) suggest the presence of ruminant fats or aquatic products ([40]) (S5 File).

### 3.2. Plant resources

#### • The contribution of cereals and grasses

**Fig 7:**
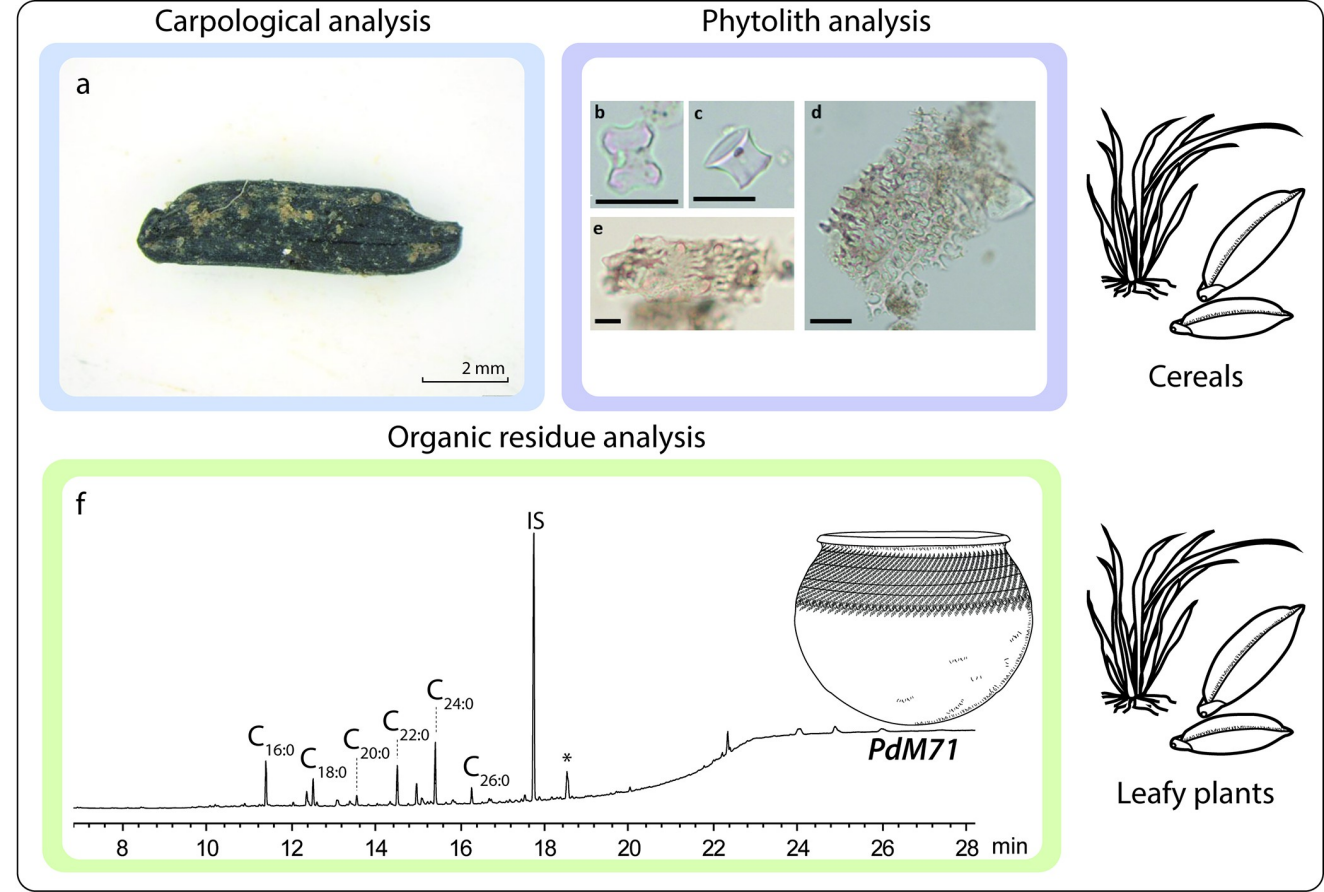
Evidence of plant consumption at the Poubelle des Mamans site: a) charred rice, b) scooped bilobate GSSCP; c) Rondel GSSCP; d) Elongate dendritic; e) Papillate.phytoliths identified in pottery (scale 20 μm) and f) chromatograms of lipid extracts from pot PdM 71. C_xx:x_: fatty acids; IS: internal standard; *: modern contamination.

Rice (*Oryza sp*.*)* is the dominant macroscopic plant remain, accounting for about 76% of the assemblage abundance (9 grains, 14 grain fragments and 5 spikelet base, Fig 7, S3 File and S3 Fig), and is present in two soil samples (25% ubiquity). In addition, 3 *Eleusine indica* caryopses are identified the carpological study samples. Phytoliths produced by monocotyledons grasses and herbs are represented in all pots reaching values between 3 and 16% depending on the samples (S6 Fig). Bilobate grass silica short cell phytoliths (GSSCP), mostly produced by the sub-family Panicoideae, are the best represented. Moreover, the morphotype Scooped Bilobate GSSCP, produced exclusively by the sub-family Ehrhartoideae to which rice (*Oryza sp*.) belongs, is observed in two pots (PdM 78 and PdM 138; S6 Fig). The presence of seeds is also suggested in some pots (PdM 48, PdM 90, PdM 127, PdM 51, PdM 138) by the identification of two typical morphotypes produced by grass inflorescences (Rondel GSSCP and Papillate; ICPN, 2019). ORA did not reveal any molecular profile that could be related to cereals in the ceramics studied.

#### • The contribution of oil palm tree

*Elaeis guineensis* (oil palm) endocarp fragments (16 fragments for a Minimum Number of Individuals MNI of 6) are recognised during carpological study (S3 File, S3 Fig). Arecaceae (palm family) is identified during the phytolith analysis (Fig 7 and S6 Fig) on all pot samples. Their values range from 32 and 65% of the assemblages. However, the interpretation of the phytoliths produced by the palm trees is complex. If they can represent pollution from the surrounding palm trees, they are also edible and a number of food preparations can be derived from their exploitation (palm oil, palm wine…). The specific fatty acid distribution of palm kernel oil ([43]) was not identified in the vessels studied by ORA. To our knowledge, no molecular compounds have yet been reported as biomarkers for other palm products such as oil extracted from the pulp, wine, or leaves.

*Other plant products*. We note in several phytolith assemblages the presence of morphotypes produced by Cyperaceae or Commelinaceae herbs. Similar to the high proportion of tree and palm phytoliths, the presence of these adventice morphotypes questions the origin and interpretation of these phytoliths, which seem more likely to come from the surrounding vegetation of the site.

One vessel (PdM 71) yielded a typical plant wax profile from leafy plants ([9, 44]): low lipid content (10.0 μg g^-1^) and a high proportion of long-chain fatty acids (LCFA; C_20:0_-C_26:0_; Fig 7f). The presence of LCFA in smaller amounts in six other sherds shows that some vessels contained plant products mixed with other commodities (PdM 3, PdM 48, PdM 49, PdM 51, PdM 78, PdM 90). Large amounts of C_16:0_ and C_18:1_ in certain vessels may also originate from plant products (or aquatic products), particularly in PdM 71, PdM 111, PdM 120 and PdM 138 (S5 File and S5.2 in S5 Fig).

### 3.3. Food preparation methods

#### • First steps of food preparation

Cutmarks can be observed on 1/10 of the collected bones and are found on most of the identified species (mammals, fish, birds, reptiles, lagomorphs and rodents), indicating the use of knives in the preparation of the animals. The carcasses of larger species, such as pigs and cattle, yielded additional percussion marks (Fig 6b). The botanical remains from the site are systematically charred (Fig 7a), indicating fire transformation (for long-term preservation purposes or as a step in a recipe), although accidental firing cannot be excluded at this stage.

#### • Cooking in pots

**Fig 8:**
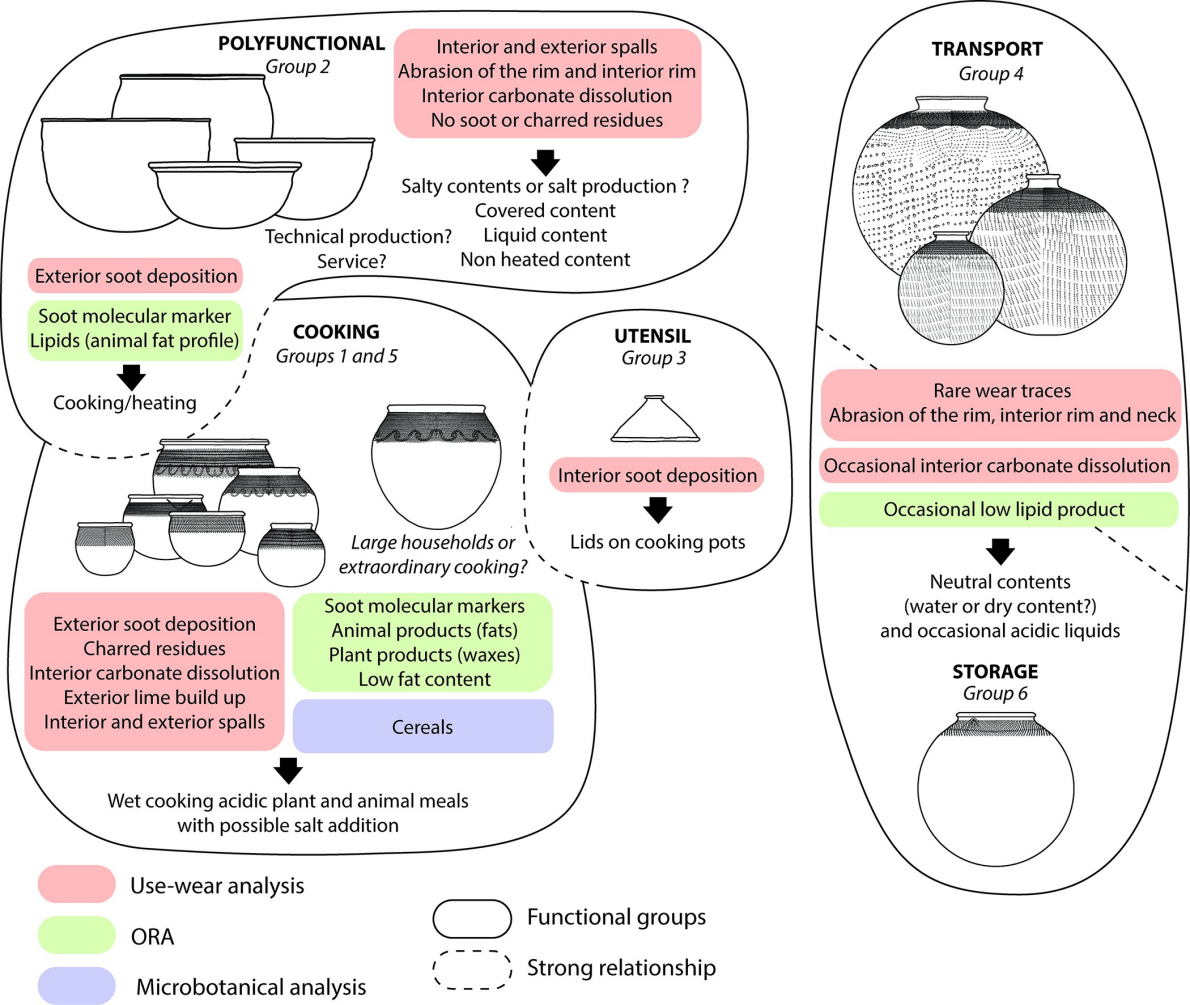
Functional groups of pottery retrieved in the Poubelle des Mamans, based on the combination of morphometry, use-alteration, ORA and microbotanical analysis.

Group 1 consists of small to medium sized pots with a restricted to slightly restricted mouth and twisted cord roulette impression (TCR), while Group 5 consists of large pots with a slightly restricted mouth (Fig 8, S4 File and S4.1 in S4 Fig). Pots from Group 5 can be compared to Group 1 and/or Group 6, as they are similar in shape and decoration, but differ in volume. Differences in volume may reflect either different functions or greater needs for the same activity ([45]).

Use-alteration traces and ORA argue for similar functions between Groups 1 and 5 (S4 File, S4.4 and S4.5 in S4 Fig). Both groups were used over fire, probably for cooking activities, as evidenced by the macroscopically visible soot and charred residues and the soot molecular markers (polyaromatic hydrocarbons) detected in half of the pots in ORA (S1 Table). Three pots from Group 5 and two from Group 1 yielded significant amounts of lipids, with typical animal fat profiles (including one with possible aquatic fat; S1 Table, S5 File). The phytolith study of one pot suggests the presence of *Oryza sp*. while the phytolith assemblages of 4 other pots indicate the presence of grass inflorescence. The scarcity of fire marks on bones may indicate that meat was preferably cooked in pots (boiling, simmering etc.) rather than roasted over an open fire. The presence of long-chain fatty acids in most of these vessels (S1 Table) suggests that animal foods were mixed with plant products (leaves, stems, seeds), although independent and successive uses cannot be ruled out. The remaining analysed vessels from these two groups preserved little lipid, with the occasional presence of long chain fatty acids, suggesting low-fat contents of plant origin.

The high frequency of dissolved carbonate temper in Groups 1 and 5 shows that liquid and acid preparations were frequent (S4 File, S4.2, S4.4 and S4.5 in S4 Fig). This dissolution led to lime precipitation on the upper part of the vessel on the outer wall due to capillarity ([46]). This observation could be made because the entire pottery assemblage is shell tempered. Temper dissolution wear can provide distinct filling limits (S4 File, S4.2 in S4 Fig), indicating liquid contents. When the dissolution is coupled with soot deposits, it is possible to interpret wet cooking techniques. Similarly, the deposition of carbonised residues in a ring shape of varying width around the filing limit (S4 File, S4.3 in S4 Fig) is indicative of wet cooking techniques ([13]). The positioning of carbonised residues at the bottom of the pot can be associated with roasting or wet cooking of highly absorbent foods, such as cereals (S4 File, S4.3 in S4 Fig; [46, 47]).

Both groups also tend to correlate with internal spalling (S4 File, S4.3 and S4.4 in S4 Fig). Several factors could lead to the formation of spalls on the inner surface of pots: 1) they could be caused by the embrittlement of the inner wall due to temper dissolution or mechanical actions that undermined the integrity of the wall ([46, 47]); 2) they can also be evidence for the presence of salt inside the pottery, as salt is absorbed in liquid form through the wall and migrates by capillarity to the upper part of the vessels where it crystallises and embrittles the surface ([47–49]).

In summary, small, medium (Group 1) and large (Group 5) pots with slightly restricted mouths were probably used as cooking pots, for a diversity of foodstuff (terrestrial animals, plants, possibly aquatic products; Fig 8). The few larger pots may reflect a larger household or occasional use for an exceptional number of mouths to feed, as can be the case during feasts, or exceptional products to cook, as can be the case for large animal parts (e. g. heads, and limbs of large animals). Although culinary activity remains the primary hypothesis, it cannot be excluded that some vessels were used for other purposes. For example, one vessel could have been used to boil medicinal leaves (exclusive leaf plant signal), a hypothesis also mentioned to interpret the presence of plant wax in Nok pottery from Nigeria ([44]).

Group 3 combines several types of undecorated largely open mouth vessels, the majority of which are open-cone in shape and have a prehensile element (Fig 8). They show a significant correlation with soot deposition on the inner wall, which is otherwise absent in the other shapes (S4.4 in S4 Fig), and suggest that they could have been used as lids for cooking pots.

#### • Transport and storage using pottery

Group 4 consists of the largest vessels in the assemblage and the smallest ratio of mouth diameter to maximum diameter (S4 File, S4.1 in S4 Fig). They are the only vessels decorated all over the body with a rolled shells impression on the lower part and a twisted cord roulette impression on the upper part. Group 6 is similar in shape, but has a wider aperture ratio than Group 4 and no rolled shell ornamentation (S4 File, S4.1 in S4 Fig). In both groups, the content did not cause heavy alteration, except for the anecdotal presence of carbonate temper dissolution on the inner surface (S4 File, S4.4 in S4 Fig). Abrasions located on the rim and inner rim correlate with each shape but are more frequent on Groups 4 and 6. They could be caused by closure systems such as plugs and/or repeated removal of content. The morphometry and use-alteration traces suggest that both shapes could have been used to hold liquids. Variability in temper dissolution suggests either a different life span of the pots or a difference in the pH of the liquid contained. The difference in aperture ratio of Group 4 and Group 6 could indicate liquid transport in Group 4 as opposed to more static storage pots in Group 6 ([27] p. 633). The pot from Group 6 analysed by ORA contained a very low-fat product, perhaps water, juice or fermented beverage (S1 Table, S5 File). However, the lipid profile extracted from Group 4 pot remains difficult to interpret due to probable contamination (S5 File).

#### • Other activities

Group 2 consists of medium-sized, open-mouthed, undecorated pots, which tend to correlate with internal and external spalling (S4 File, Fig 8). The majority of other alterations are also found: abrasions of the rim and inner rim, temper dissolution (S4 File, S4.4 in S4 Fig). The lack of correlation with charred residues and soot suggests that most of them were not used in any activity over fire. However, some were probably used as cooking vessels (3 pots with macroscopic and molecular traces of soot), of which at least one was used for cooking fatty animal products, very similar to some vessels from Groups 1 and 5 (S1 Table). Some traces, such as spalls on the inner surface of the pots may be related to salt production activities and should be investigated further. The analysis of phytoliths does not provide a clear interpretation. In short, Group 2 appears to be a group of polyfunctional vessels.

## 4. Reconstructing foodways at the Poubelle des Mamans

**Fig 9:**
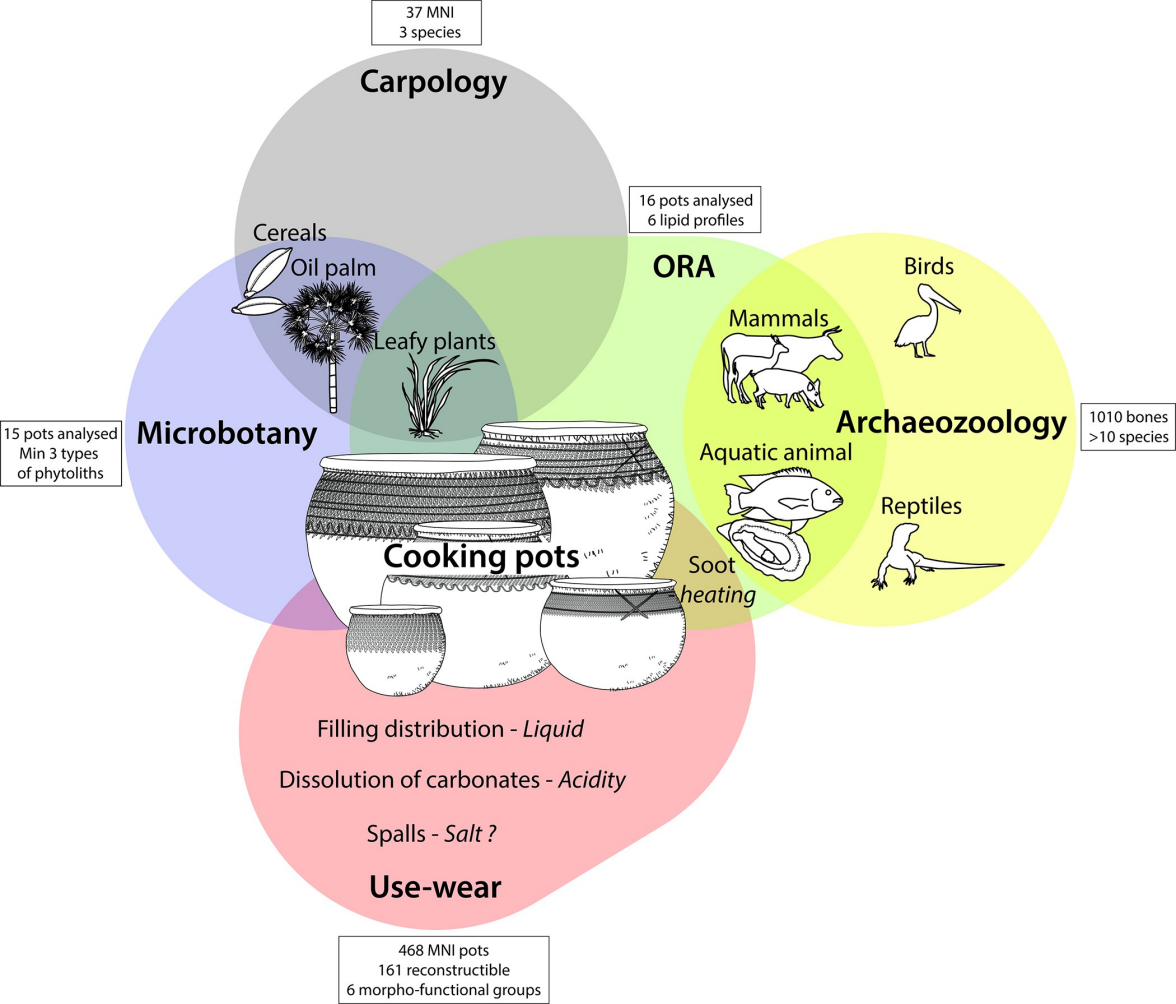
Synthesis of information on foodways in Edioungou using an interdisciplinary approach.

The multidisciplinary and integrated study of this dump site provides a first picture of the foodways at Edioungou during the 20th century (Fig 9). The use of different archaeological disciplines has partly compensated for the taphonomic effects and limitations of each approach: for example, while there is no molecular biomarker for rice in organic residue analysis, phytoliths provided information on its presence in pots. The implementation of a common and concerted sampling and analysis strategy, prior to the studies in each discipline resulted in a large amount of information on foodways that could be correlated for further discussion. In particular, the potsherd analysis strategy was designed to reconcile the constraints of each discipline: the morphometric study on unwashed sherds has allowed a sampling of vessels for microbotanical analysis and ORA, in line with our research questions. These analyses were carried out at the same level on the pot based on the preliminary use alteration analysis. Finally, the use-alteration study was carried out on washed sherds to help establish functional groups.

The diet appears to be mainly based on rice (carpological analysis) and the consumption of fish, pigs, cattle, and oysters (archaeozoological and ichthyological analysis and some ORA data). Other foods contribute to the diet to a lesser extent: poultry and game species (archaeozoology), oil palm products, panicoid cereals such as maize, sorghum and millet (phytoliths) and undetermined leafy plants (ORA). Cooking vessels with restricted or slightly restricted mouths and a wide range of volumes were used to cook, by boiling or simmering (use-alteration analysis), various animal (terrestrial mammals and aquatic products) and vegetable (leafy plants and cereals) foods. A large proportion of these vessels are characterised by acidic and possibly salty contents (use-alteration analysis), including those that have been used for animal products, suggesting possible food mixtures as part of cooking recipes. Cooking activities are well represented in the pottery assemblages through a diversity of shapes and sizes, which may reflect the variety of cooked preparations. The larger size cooking pots can be associated with large household or exceptional meals (feasts and/or large animal parts). Although cooking pots were probably used for culinary purposes, medicinal uses cannot be excluded. Ceramic containers for storage and transport are mainly associated with contents that are neither acidic, salty nor fermented, either dry goods or neutral liquids, and with abrasion caused by plug systems or content removal (use-alteration analysis). The very narrow mouths argue for the second option. However, when wear is observed on this group of vessels, it shows specific traces of acidic and non-fatty liquids. The use of more or less acidic foods seems to be a characteristic of Diola Kassa cuisine (use-alteration analysis), and some containers may have had very salty contents, although this remains to be investigated.

Further investigation using the disciplines introduced in this paper should provide a more complete picture of food practices, particularly with regard to preparation and consumption patterns. The detailed study of butchery traces should provide information on cutting methods, choice of parts consumed and the distribution practices of animal parts. A study of the carbonised residues found in the sediment, by microscopic observation, scanning electron microscope (SEM), lipid and phytolith extraction, should confirm whether they are food residues and identify possible mixtures. They could also provide information on how certain foods were prepared (e.g. cereals eaten as porridge, bread, etc.; e.g. [50, 51]). Such analyses could also be used to check whether the palm and adventice phytoliths found in the pots originate from food or from contamination by waste or surrounding plants (roots, leaves, etc.). The extraction of small water-soluble acids from plants ([22]) could provide additional information on the plant foods consumed (e.g. fruits, fermented beverages) and contribute to the discussion of the nature of the foods that produced the acidity responsible for temper dissolution. The continuation of the study of spalls on the pottery surface will also assess the production of salt and its use in food preparation.

We cannot exclude the possibility that disposal practices and degradation processes have in part biased our interpretation of food practices at the site. For example, service and consumption pots are not clearly identified and may be absent or marginal in the assemblage. These functions could be fulfilled by organic containers such as wooden vessels or calabashes, or this absence could imply a lack of need for such functional categories as meals and beverages could be eaten directly from the cooking pot. The transport and storage pots of the assemblage seem to be more liquid-oriented (narrow mouths, plug marks, acidic liquids) which raises the question of how dry goods were stored, possibly in organic containers (baskets, bags…) or in granaries. It should also be borne in mind that some of the foodstuffs leave very few archaeological remains (e.g. vegetables, seasoning), and that food practices may not have involved preparation in pottery or exposure to heat, which does not favour their identification. For example, the consumption of raw or steamed foods (e.g. fruit, vegetables, meat, fish) is not documented at the site, perhaps due to the limited evidences left by such practices. In addition, the absence of fish scales at the site suggests that some of the discarding of food preparations rebus took place elsewhere in or outside the village. This means that some of the dietary practices may not be represented at the dump site and therefore escape our picture.

In order to verify and complement the interpretations derived from the study of the Poubelle des Mamans and to identify possible biases in our archaeological approach, we will compare our results with those of the ethnoarchaeological study of pottery functions and food practices carried out in the village of Edioungou. This strategy will provide a better understanding of the material expressions of food behaviours and investigate the diachronic evolution of food practices over about a century.

## Supporting information

S1 Fig C dating of two charcoals from the lowest unit of the excavation (dec 5), resting on the *substratum*. (TIF)

S2 Fig **1.** Number, weight and frequency of hand-collected faunal remains collected in "La Poubelle des Mamans", by species, species category or unspecified. **2.** Elements from the sieved samples and extracted ichthyological material (right).
(ZIP)

S3 Fig Summary of the archaeobotanical results. Total numbers, frequency and ubiquity of identified items summarised according to chronological contexts.
(TIF)

S4 Fig **1.** Scatter graph showing the morphological groups of the pottery assemblage according to height and percentage of aperture. **2.** Addition traces from the site of La Poubelle des Mamans. **3.** Subtractive wears from the site of La Poubelle des Mamans. **4.** Correspondence Analysis of use-alteration traces according to morpho-stylistic analysis. a) results with Groups 1, 2, 3 (lids), 4, 5 and 6. Pearson’s Chi squared test = 185.1599, p-value = 5.374969e-16; b) results without Group 3 (lids). Pearson’s Chi squared test = 77.88525, p-value = 0.000311663. **5.** Matrigraph of the representation of use-alterations according to morpho-stylistic groups providing detailed quantitative data.
(ZIP)

S5 Fig **2.** Lipid yield and fatty acid ratios in the samples studied in organic residue analysis. **3.** Single compound stable carbon isotopes in lipid extracts. a) δ^13^C_18:0_ values plotted against δ^13^C_16:0_ values. The 95% confidence ellipses are calculated using authentic reference fat values published in the literature [3, 11–24]. b) Δ^13^C values plotted against their δ^13^C_16:0_ value. **4.** Chromatograms of lipid extracts from pot PdM 111. a) after DCM/MeOH extraction; b) after acid transmethylation (SIM mode analysis). C_xx:x_: fatty acids; C_xx:x_br: branched fatty acids; TAGs: triacylglycerols; IS: internal standard.
(ZIP)

S6 Fig Phytolith diagram of the 15 pots of La Poubelle des Mamans. (TIF)

S1 Table Extraction yields, molecular compositions and stable carbon isotope values in samples studied by ORA. (XLSX)

S1 File Excavation and material. (DOCX)

S2 File Archaeozoology. (DOCX)

S3 File Carpology. (DOCX)

S4 File Morphometrical and use-wear approaches to pottery. (DOCX)

S5 File Organic residue analysis. (DOCX)

S6 File Microbotanical remains. (DOCX)
