# Supplementary figures and images for: Investigating grandmothers’ cooking: A multidisciplinary approach to foodways on an archaeological dump in Lower Casamance, Senegal
Source: PLoS One. 2024 May 29;19(5):e0295794. doi: 10.1371/journal.pone.0295794 (PMC11135772; doi:10.1371/journal.pone.0295794)

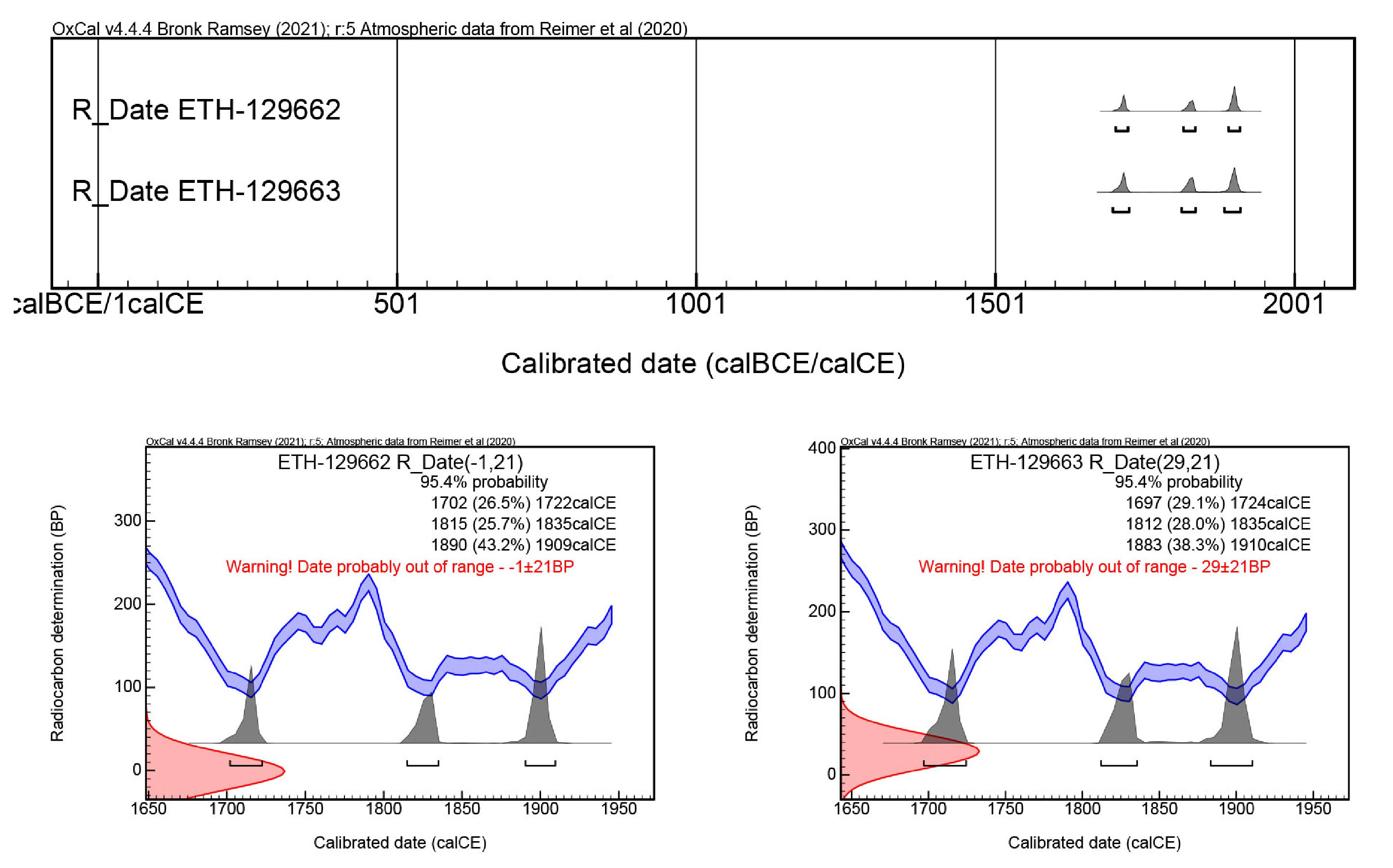

Supplement: S1 Fig — (TIF) [file pone.0295794.s001.tif]

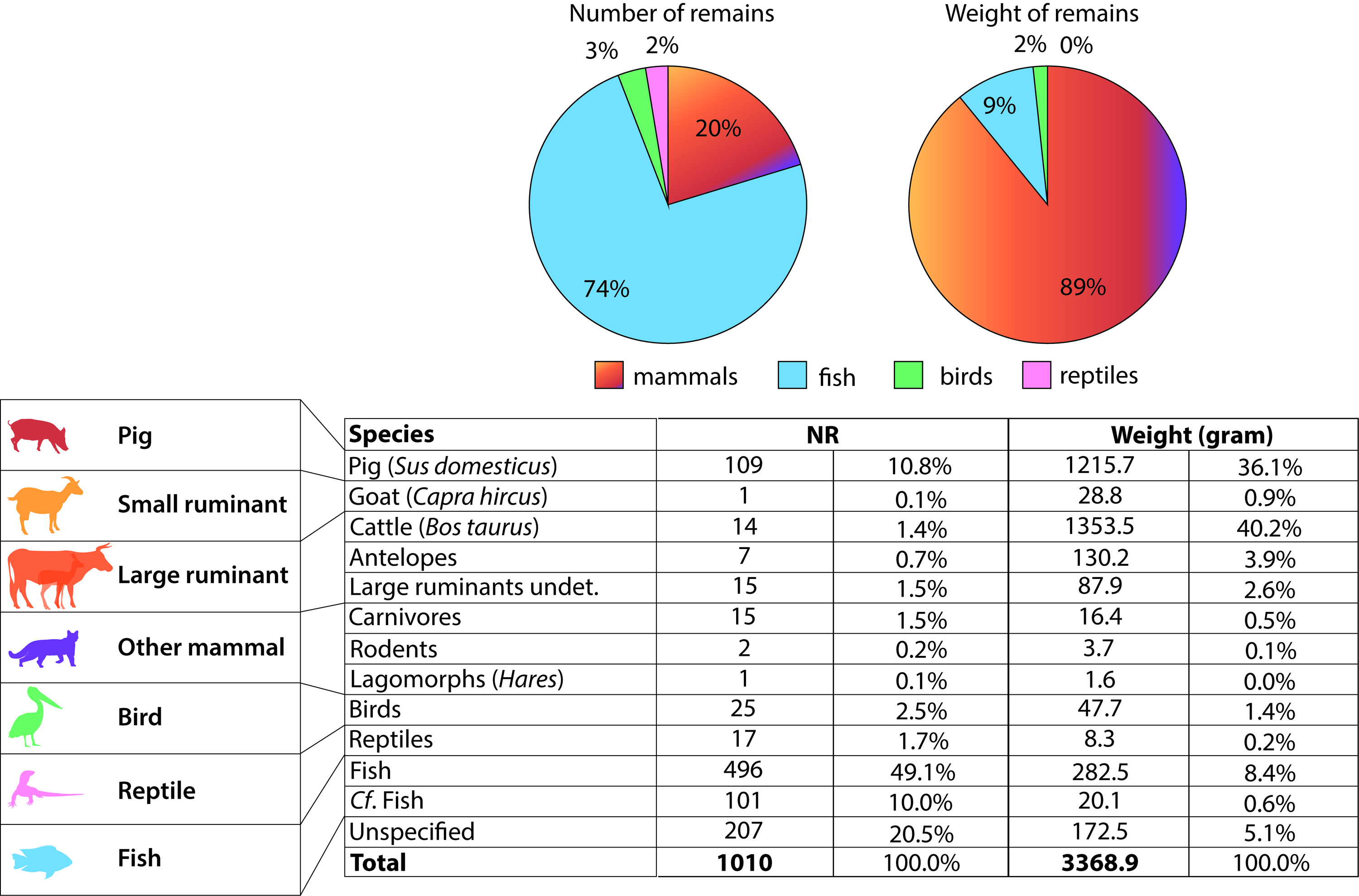

Supplement: S2 Fig — 1. Number, weight and frequency of hand-collected faunal remains collected in "La Poubelle des Mamans", by species, species category or unspecified. 2. Elements from the sieved samples and extracted ichthyological material (right). (ZIP) [file pone.0295794.s002.zip › S2.1_fig.tif]

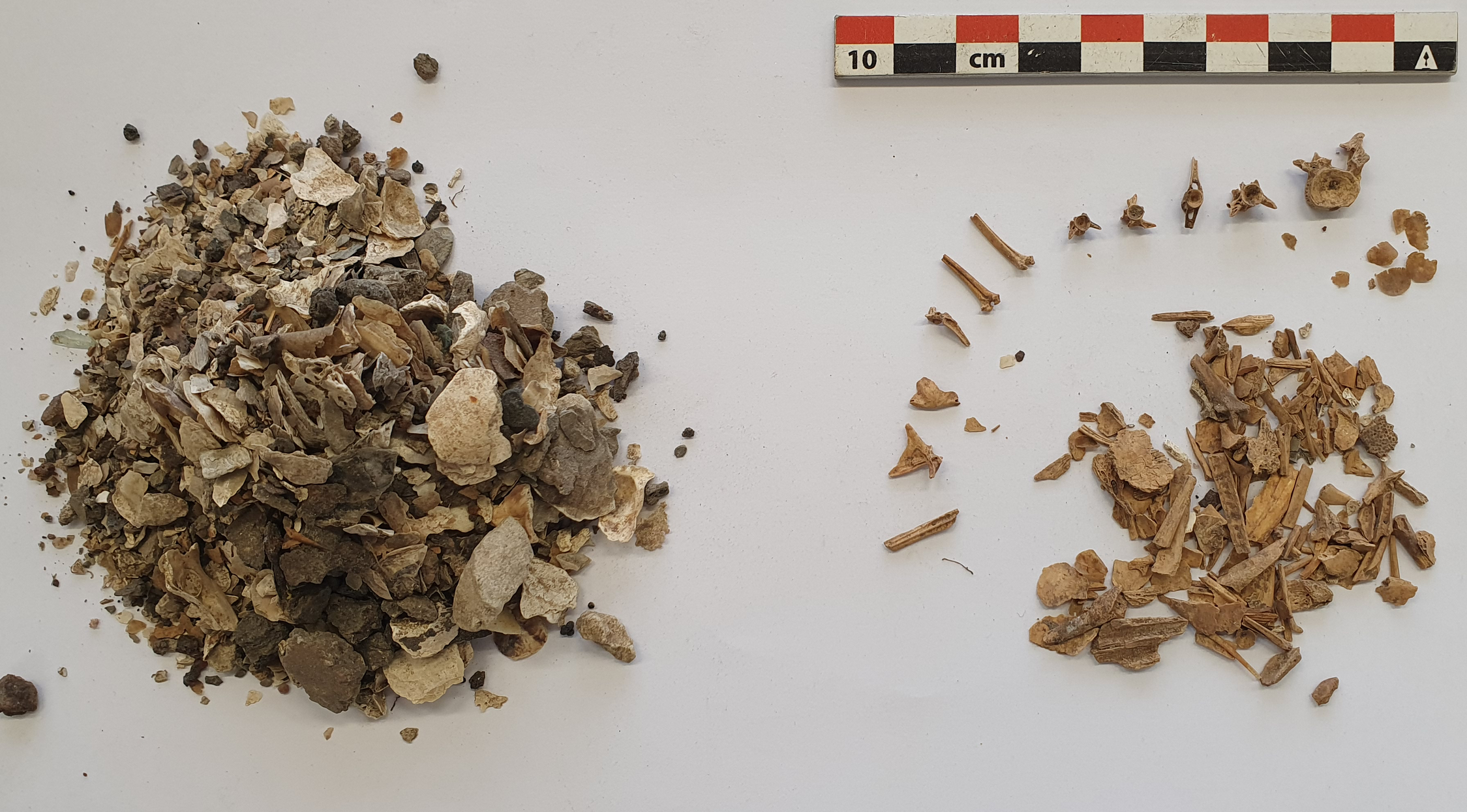

Supplement: S2 Fig — 1. Number, weight and frequency of hand-collected faunal remains collected in "La Poubelle des Mamans", by species, species category or unspecified. 2. Elements from the sieved samples and extracted ichthyological material (right). (ZIP) [file pone.0295794.s002.zip › S2.2_fig.tif]

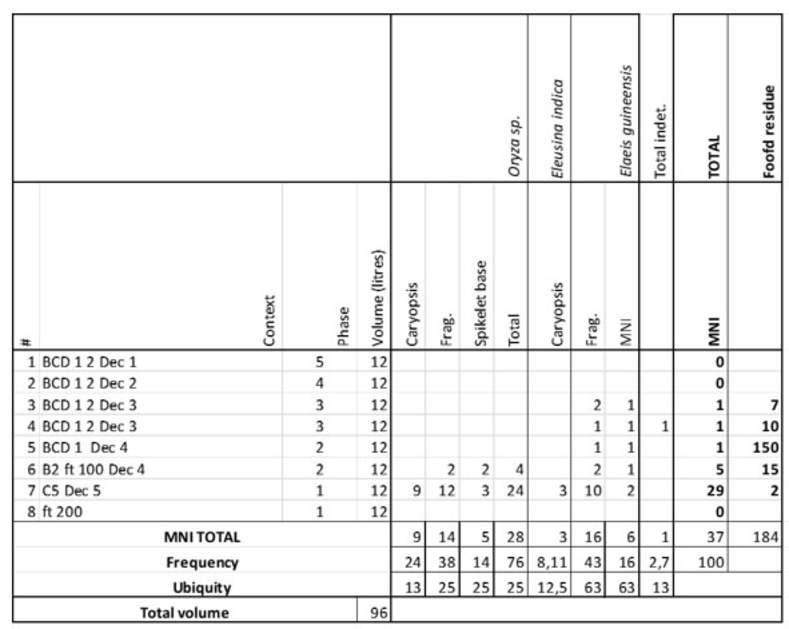

Supplement: S3 Fig — Total numbers, frequency and ubiquity of identified items summarised according to chronological contexts. (TIF) [file pone.0295794.s003.tif]

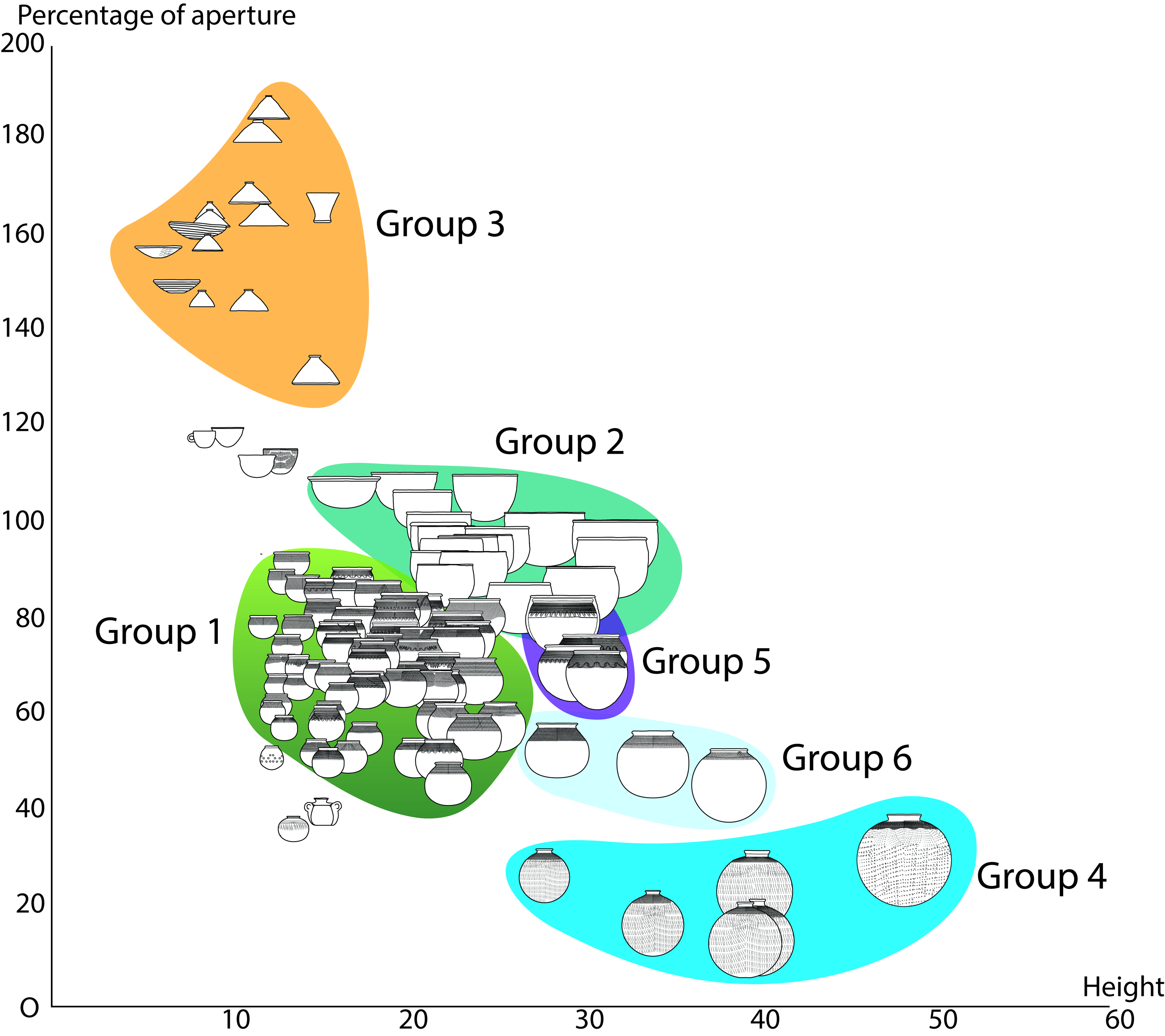

Supplement: S4 Fig — 1. Scatter graph showing the morphological groups of the pottery assemblage according to height and percentage of aperture. 2. Addition traces from the site of La Poubelle des Mamans. 3. Subtractive wears from the site of La Poubelle des Mamans. 4. Correspondence Analysis of use-alteration traces according to morpho-stylistic analysis. a) results with Groups 1, 2, 3 (lids), 4, 5 and 6. Pearson’s Chi squared test = 185.1599, p-value = 5.374969e-16; b) results without Group 3 (lids). Pearson’s Chi squared test = 77.88525, p-value = 0.000311663. 5. Matrigraph of the representation of use-alterations according to morpho-stylistic groups providing detailed quantitative data. (ZIP) [file pone.0295794.s004.zip › S4.1_fig.tif]

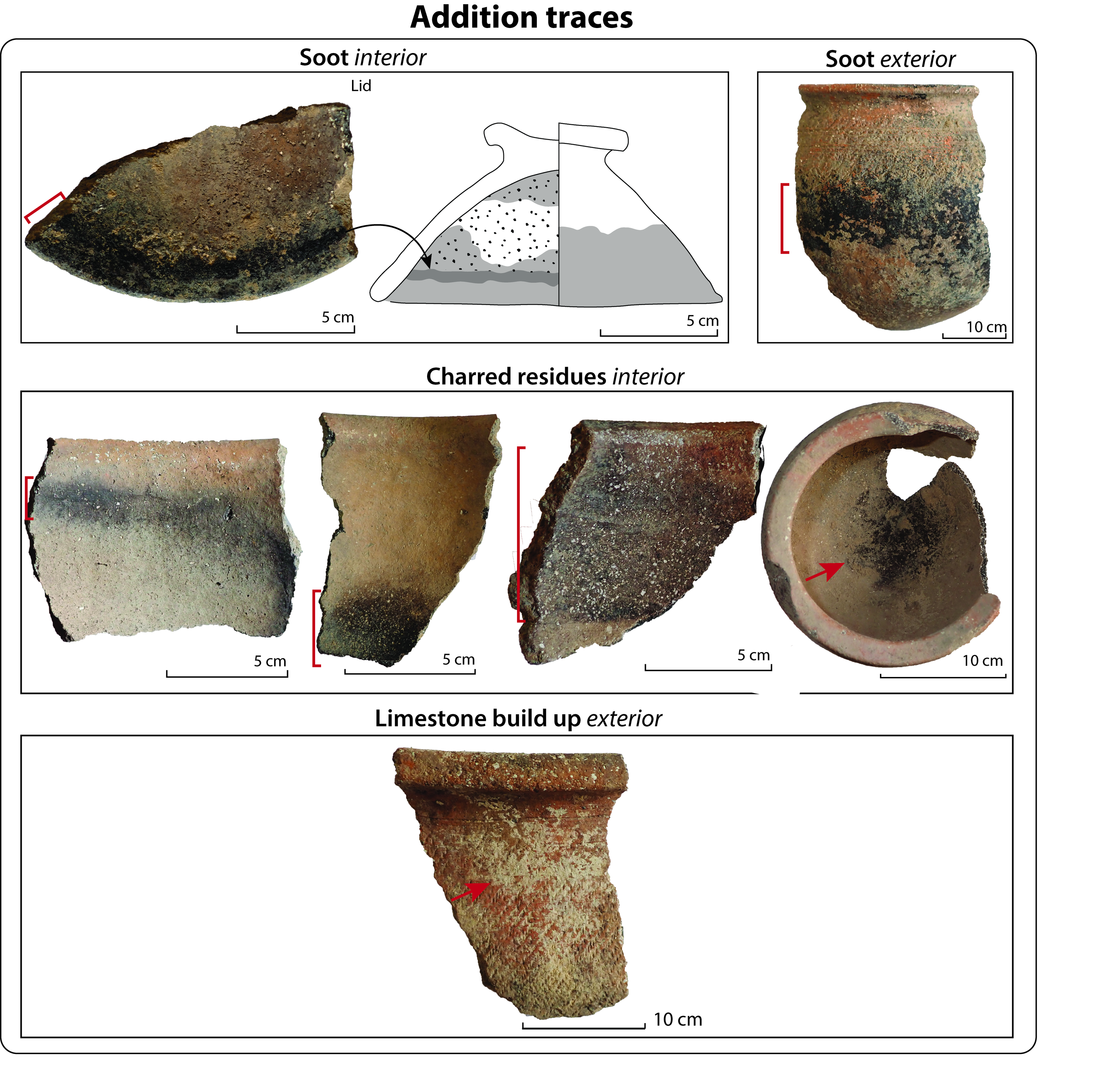

Supplement: S4 Fig — 1. Scatter graph showing the morphological groups of the pottery assemblage according to height and percentage of aperture. 2. Addition traces from the site of La Poubelle des Mamans. 3. Subtractive wears from the site of La Poubelle des Mamans. 4. Correspondence Analysis of use-alteration traces according to morpho-stylistic analysis. a) results with Groups 1, 2, 3 (lids), 4, 5 and 6. Pearson’s Chi squared test = 185.1599, p-value = 5.374969e-16; b) results without Group 3 (lids). Pearson’s Chi squared test = 77.88525, p-value = 0.000311663. 5. Matrigraph of the representation of use-alterations according to morpho-stylistic groups providing detailed quantitative data. (ZIP) [file pone.0295794.s004.zip › S4.2_fig.tif]

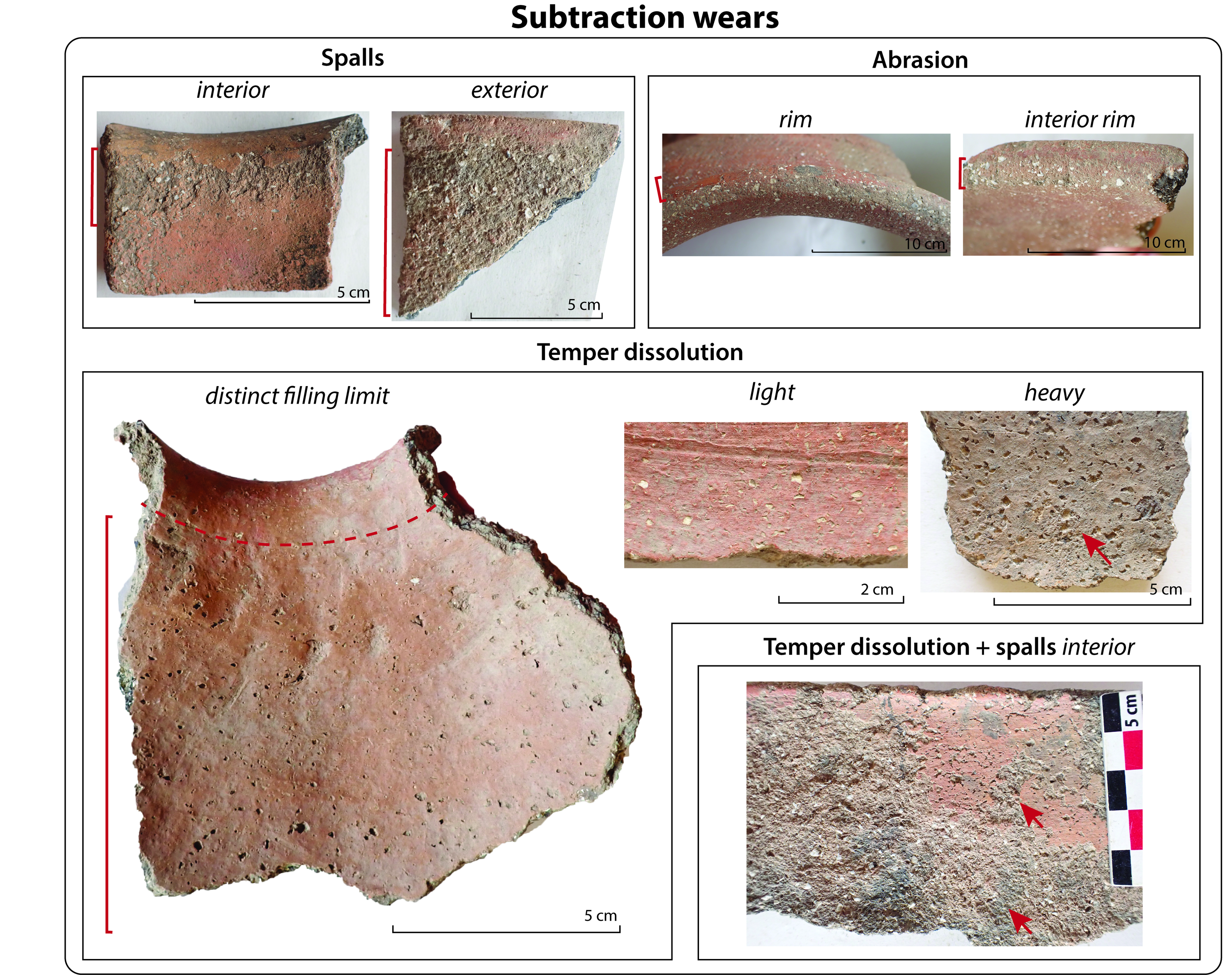

Supplement: S4 Fig — 1. Scatter graph showing the morphological groups of the pottery assemblage according to height and percentage of aperture. 2. Addition traces from the site of La Poubelle des Mamans. 3. Subtractive wears from the site of La Poubelle des Mamans. 4. Correspondence Analysis of use-alteration traces according to morpho-stylistic analysis. a) results with Groups 1, 2, 3 (lids), 4, 5 and 6. Pearson’s Chi squared test = 185.1599, p-value = 5.374969e-16; b) results without Group 3 (lids). Pearson’s Chi squared test = 77.88525, p-value = 0.000311663. 5. Matrigraph of the representation of use-alterations according to morpho-stylistic groups providing detailed quantitative data. (ZIP) [file pone.0295794.s004.zip › S4.3_fig.tif]

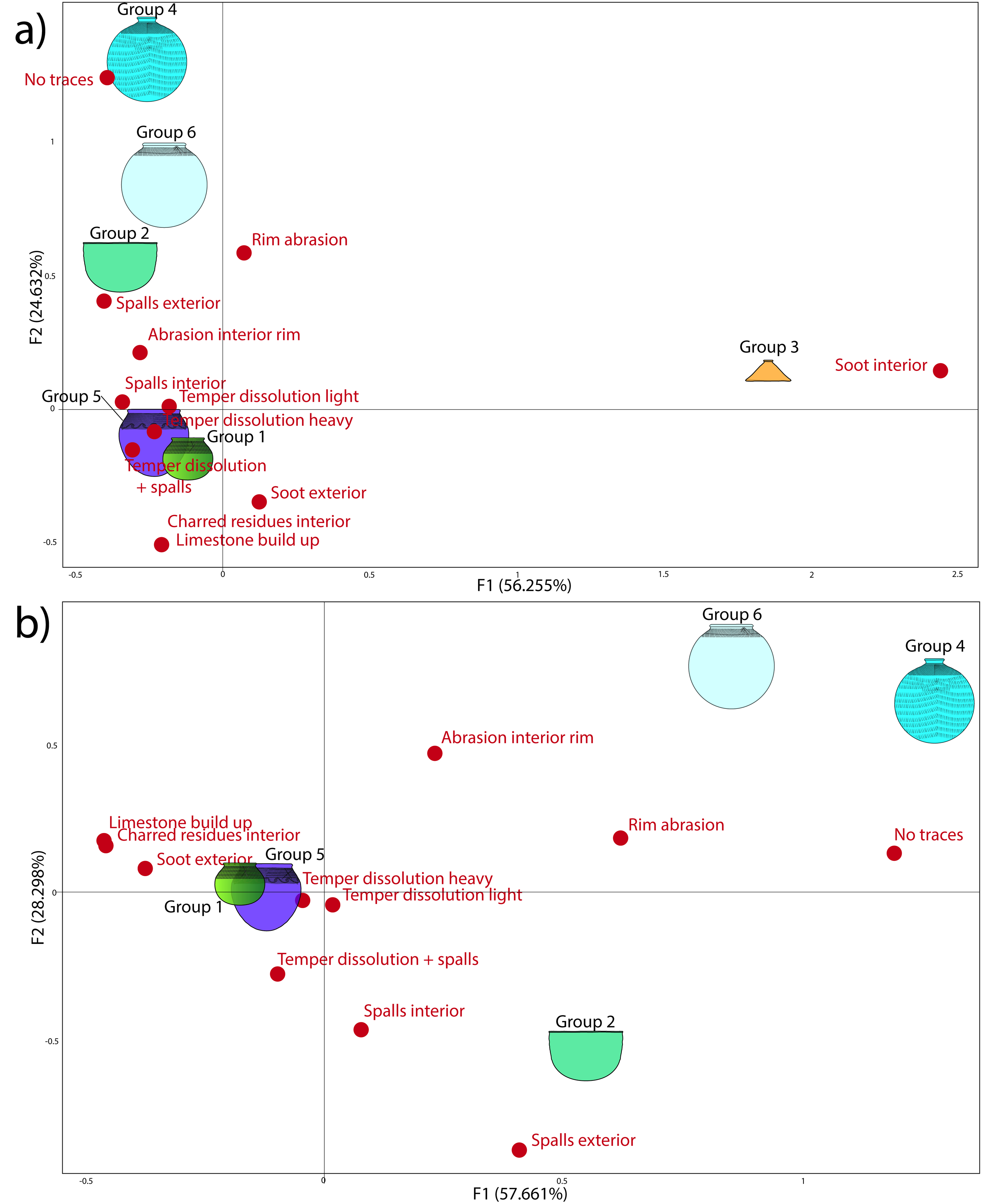

Supplement: S4 Fig — 1. Scatter graph showing the morphological groups of the pottery assemblage according to height and percentage of aperture. 2. Addition traces from the site of La Poubelle des Mamans. 3. Subtractive wears from the site of La Poubelle des Mamans. 4. Correspondence Analysis of use-alteration traces according to morpho-stylistic analysis. a) results with Groups 1, 2, 3 (lids), 4, 5 and 6. Pearson’s Chi squared test = 185.1599, p-value = 5.374969e-16; b) results without Group 3 (lids). Pearson’s Chi squared test = 77.88525, p-value = 0.000311663. 5. Matrigraph of the representation of use-alterations according to morpho-stylistic groups providing detailed quantitative data. (ZIP) [file pone.0295794.s004.zip › S4.4_fig.tif]

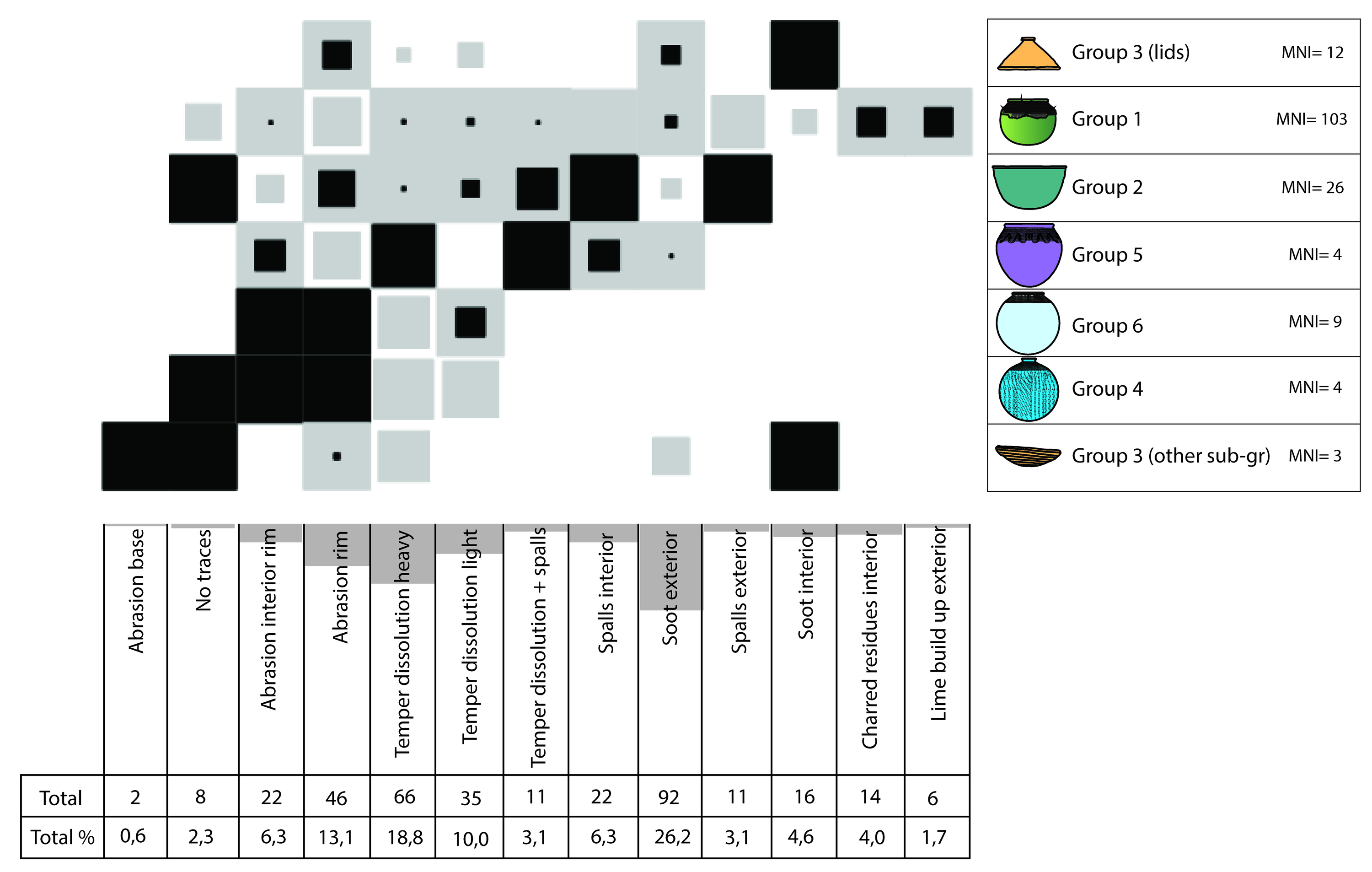

Supplement: S4 Fig — 1. Scatter graph showing the morphological groups of the pottery assemblage according to height and percentage of aperture. 2. Addition traces from the site of La Poubelle des Mamans. 3. Subtractive wears from the site of La Poubelle des Mamans. 4. Correspondence Analysis of use-alteration traces according to morpho-stylistic analysis. a) results with Groups 1, 2, 3 (lids), 4, 5 and 6. Pearson’s Chi squared test = 185.1599, p-value = 5.374969e-16; b) results without Group 3 (lids). Pearson’s Chi squared test = 77.88525, p-value = 0.000311663. 5. Matrigraph of the representation of use-alterations according to morpho-stylistic groups providing detailed quantitative data. (ZIP) [file pone.0295794.s004.zip › S4.5_fig.tif]

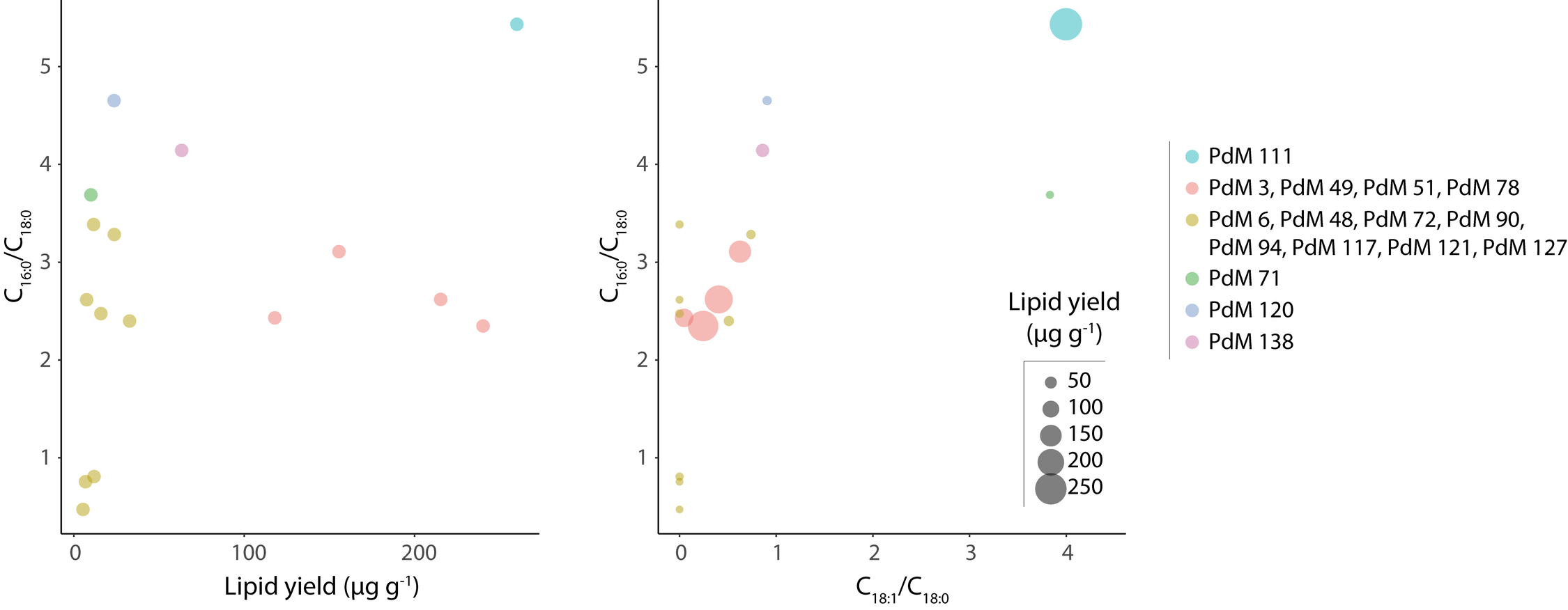

Supplement: S5 Fig — 2. Lipid yield and fatty acid ratios in the samples studied in organic residue analysis. 3. Single compound stable carbon isotopes in lipid extracts. a) δ13C18:0 values plotted against δ13C16:0 values. The 95% confidence ellipses are calculated using authentic reference fat values published in the literature [3, 11–24]. b) Δ13C values plotted against their δ13C16:0 value. 4. Chromatograms of lipid extracts from pot PdM 111. a) after DCM/MeOH extraction; b) after acid transmethylation (SIM mode analysis). Cxx:x: fatty acids; Cxx:xbr: branched fatty acids; TAGs: triacylglycerols; IS: internal standard. (ZIP) [file pone.0295794.s005.zip › S5.2_fig.tif]

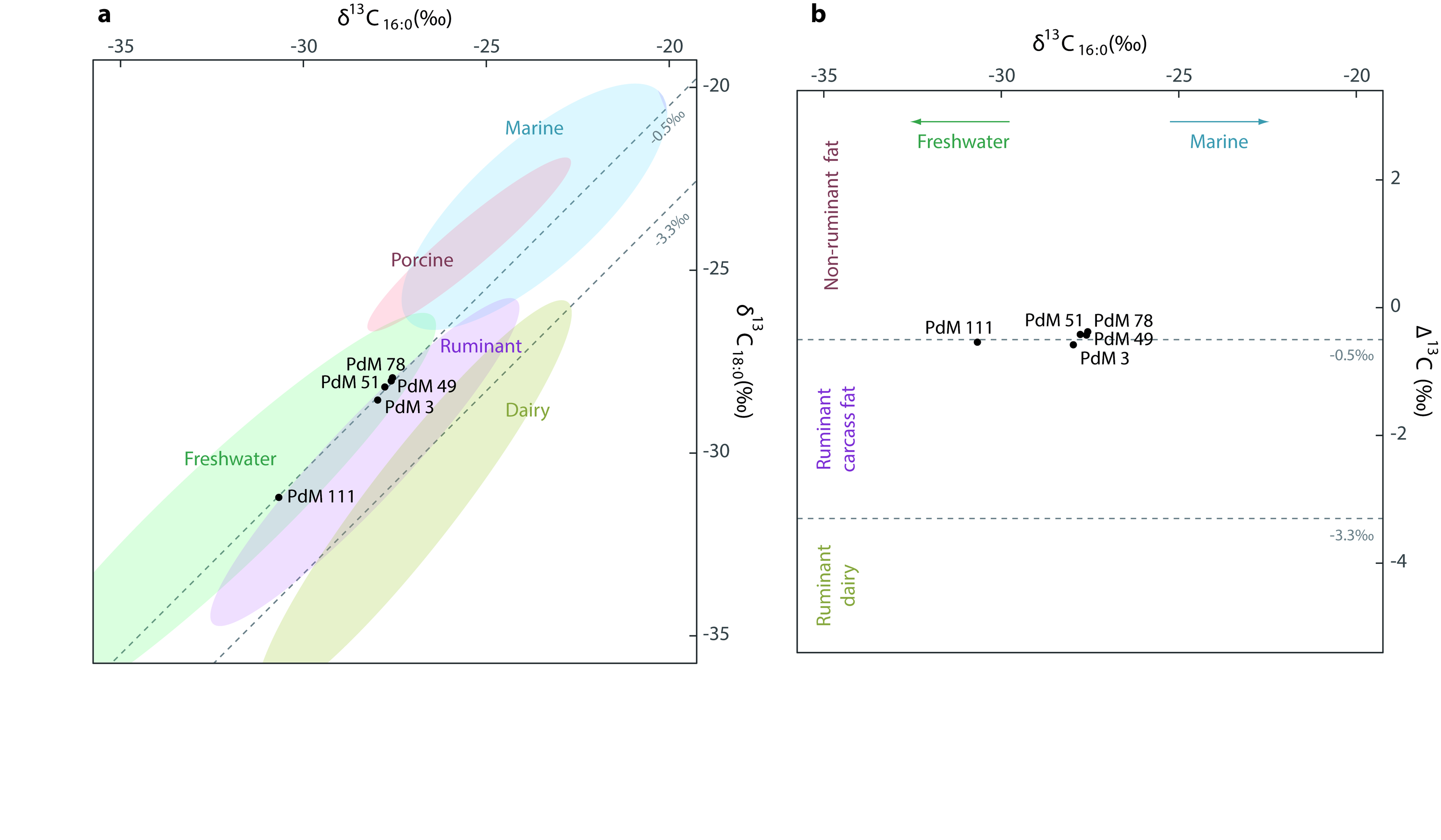

Supplement: S5 Fig — 2. Lipid yield and fatty acid ratios in the samples studied in organic residue analysis. 3. Single compound stable carbon isotopes in lipid extracts. a) δ13C18:0 values plotted against δ13C16:0 values. The 95% confidence ellipses are calculated using authentic reference fat values published in the literature [3, 11–24]. b) Δ13C values plotted against their δ13C16:0 value. 4. Chromatograms of lipid extracts from pot PdM 111. a) after DCM/MeOH extraction; b) after acid transmethylation (SIM mode analysis). Cxx:x: fatty acids; Cxx:xbr: branched fatty acids; TAGs: triacylglycerols; IS: internal standard. (ZIP) [file pone.0295794.s005.zip › S5.3_fig.tif]

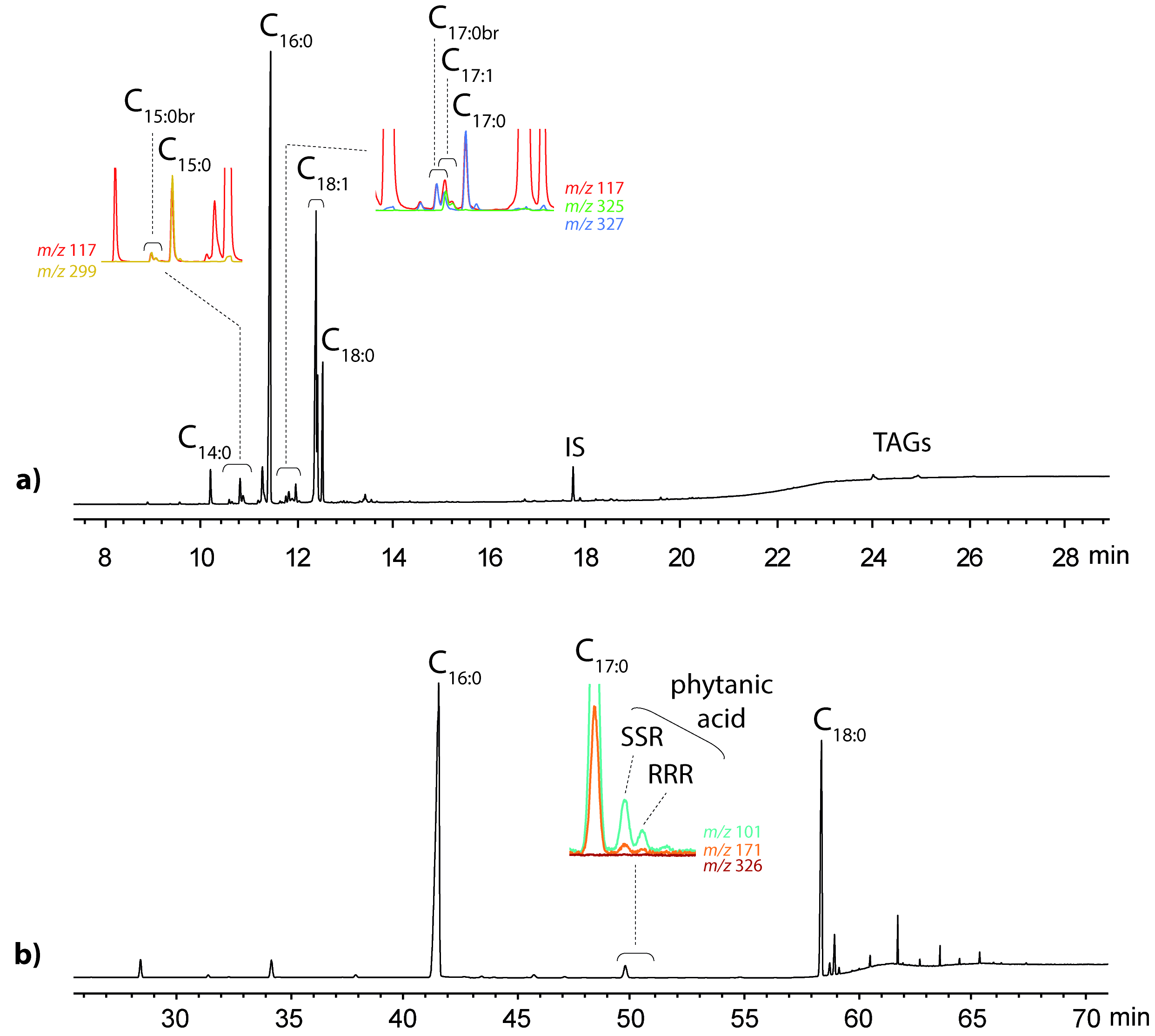

Supplement: S5 Fig — 2. Lipid yield and fatty acid ratios in the samples studied in organic residue analysis. 3. Single compound stable carbon isotopes in lipid extracts. a) δ13C18:0 values plotted against δ13C16:0 values. The 95% confidence ellipses are calculated using authentic reference fat values published in the literature [3, 11–24]. b) Δ13C values plotted against their δ13C16:0 value. 4. Chromatograms of lipid extracts from pot PdM 111. a) after DCM/MeOH extraction; b) after acid transmethylation (SIM mode analysis). Cxx:x: fatty acids; Cxx:xbr: branched fatty acids; TAGs: triacylglycerols; IS: internal standard. (ZIP) [file pone.0295794.s005.zip › S5.4_fig.tif]

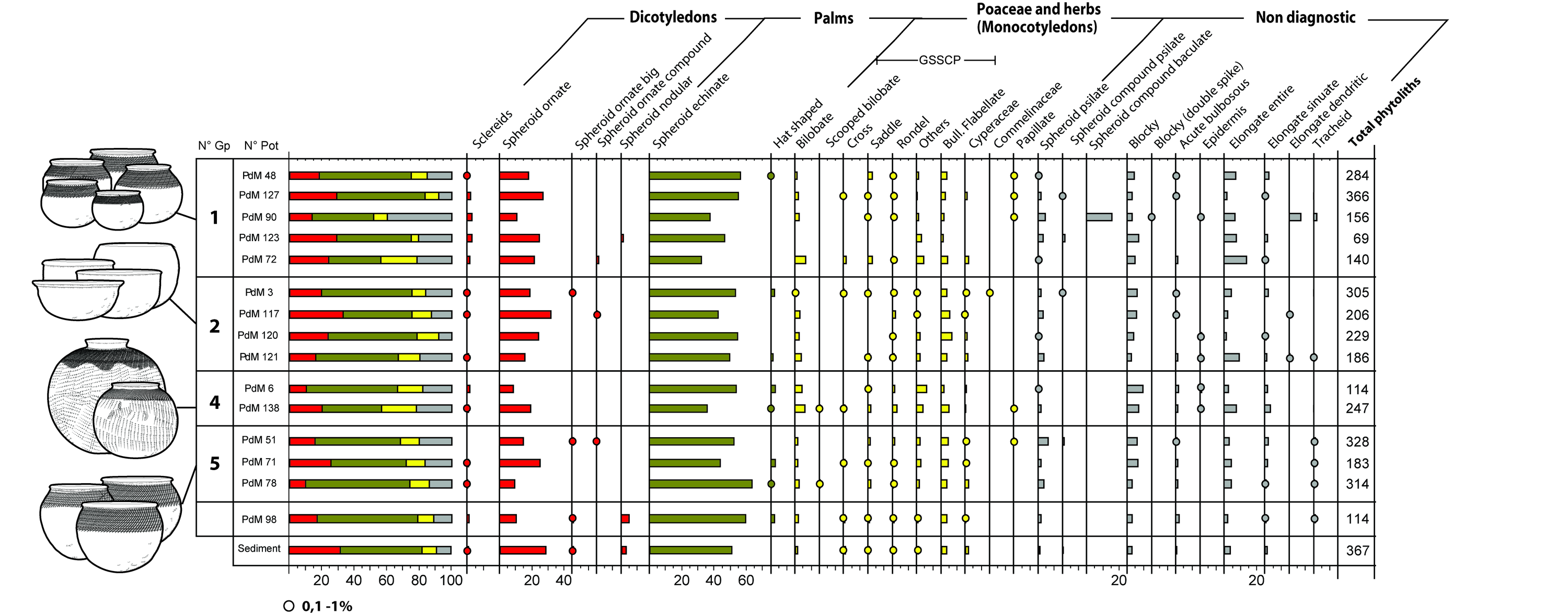

Supplement: S6 Fig — (TIF) [file pone.0295794.s006.tif]
