# Supplementary material for: Investigating grandmothers’ cooking: A multidisciplinary approach to foodways on an archaeological dump in Lower Casamance, Senegal
Source: PLoS One. 2024 May 29;19(5):e0295794. doi: 10.1371/journal.pone.0295794 (PMC11135772; doi:10.1371/journal.pone.0295794)
Supplement: S1 File — (DOCX) [file pone.0295794.s008.docx]

**S1 File - Excavation and material**

The pottery collection is curated at the IFAN (*Institut fondamental d’Afrique Noire*, Dakar). Part of this collection (≃ 20%) has been sampled and sent for further analysis to the ARCAN laboratory in Geneva (Switzerland), the LGP laboratory in Thiais (France) and the CEPAM laboratory in Nice (France), together with all the bone material and soil samples for carpological and micro-faunal analysis (ARCAN). Once all analyses have been completed, the entire collection will be curated at IFAN. Plastic and metal artefacts found during the excavation were documented on site and some of the most representative elements were sampled for conservation (IFAN), while the rest were discarded.

^14^C dating was carried out at the Laboratory of Ion Beam Physics (ETH Zurich). Charcoals were sampled from the lowest unit (dec 5). After analysis, they were identified as charred roots which required for caution in interpretation. The results show that the samples could date back from the beginning of the 18th, 19th or the beginning of the 20th century (S1.1. Fig). The most recent date is compatible with oral tradition.

**Fig S1.1: ^14^C dating of two charcoals from the lowest unit of the excavation (dec 5), resting on the *substratum*.**
