# Supplementary material for: Investigating grandmothers’ cooking: A multidisciplinary approach to foodways on an archaeological dump in Lower Casamance, Senegal
Source: PLoS One. 2024 May 29;19(5):e0295794. doi: 10.1371/journal.pone.0295794 (PMC11135772; doi:10.1371/journal.pone.0295794)
Supplement: S2 File — (DOCX) [file pone.0295794.s009.docx]

**S2 File - Archaeozoology**

**Method**

A large assemblage of mammal, fish and bird bones was collected during the excavation of “La Poubelle des Mamans”. The remains were carefully cleaned with water in the laboratory, which also allowed us to look at fresh bone fractures that had occurred during the excavation. As a result, more than a hundred reassemblies of bones were found, representing 10% of the assemblage. This timely operation had two main advantages: 1) it provided a better view of the original fractures, 2) it increased the chances of identifying pieces, which meant reducing the number of undetermined remains.

A total of eight sieved sediment samples (96 litres of soil), resulting from the flotation carried out as part of the archaeobotanical analysis, were sorted by naked eye in order to separate the bone remains and shell fragments from the other materials that make up the dump (ceramics, glass, iron, fabric/textile, etc.).

The fauna was identified to skeletal elements and species at the Museum of Natural History of Geneva (MHNG), using the collection of modern comparative skeletons for comparison (Archaeozoology Laboratory). The identification of fish remains required the creation of a new reference collection. Used for comparative osteology, a collection of modern skeletons of fish caught in July 2021 near the village of Edioungou, was the main resource used to identify the fish remains from this archaeological site. A total of 119 fish belonging to 23 species from 13 different families were purchased from two fishermen. The specimens were identified, measured, weighed and photographed in collaboration with IFAN biologists, Pr. Khady Diouf Goudiaby and engineer biologist Yacine N'diour. The fish were then boiled in water for a few minutes in water to facilitate the removal of the flesh. The skin of one side was removed in order to recover the scales. It was then bagged with salt for preservation, cleaned and processed in the MHNG laboratory. The rest of the fish was carefully gutted to facilitate the collection of all the skeletal elements. These were placed in a net with salt and dried in the open air before being stored in a plastic bag (minigrip) until further processing in the laboratory. The latter was carried out by taxidermists Jean-Marie Zumstein and Agathe Bonno, partly at IFAN, and partly at MHNG. This treatment consists of rinsing and cooking the bones in order to remove the last shreds of flesh. The bones are then dried and sorted by category (vertebrae, ribs, skull bones, other remains) and packed in boxes with an inventory number.

**Results and discussion**

Hand collected remains:

The excavation of “La Poubelle des Mamans'' yielded 1010 bone remains representing more than 3 kg of material. Considered as a whole, the spectrum shows a marked contribution from fish, representing well over half of the faunal assemblage (ca. 59% of total remains and 74% of identified remains, S2.1 Fig). Only one fish scale was found in the material collected during the excavation, i.e. identified with the naked eye. Mammals account for only 20% of the identified remains but represent 89% of their weight (S2.1 Fig). Of these, the pigs are the most important in terms of the number of remains, while cattle and carnivores are the next two most common taxa but they are present in much smaller numbers than pigs. Goat remains are rare and only one bone was identified. If we look not at the number of remains identified but at their weight, a method of quantification that better takes into account weight differences between mammals, the contribution of cattle is slightly higher than that of pigs. Numerous cutmarks are observed on a third of the determined bones, indicating the consumption of mammals, but also fish, birds, monitors, hares and rodents.

**Fig. S2.1: Number, weight and frequency of hand-collected faunal remains collected in "La Poubelle des Mamans", by species, species category or unspecified.**

Sieved samples:

Sieving of the sediments yielded over 1200 bone remains and as many shell fragments (S2.2 Fig). The vertebrate remains are largely dominated by fish. Their preservation is remarkable, as evidenced by the presence of very fragile skull parts from individuals less than 10 cm long and a few dozen scales. The many shell fragments from these samples provide a measure of the contribution of this waste to the dump. They are also a direct evidence of the contribution of this resource to the diet.

**Fig S2.2 Elements from the sieved samples and extracted ichthyological material (right).**

These first results clearly show the complementarity of the two types of sampling (hand-collected remains and after sieving): while both attest to the consumption of large fish (>30cm in length), the importance of mammals, especially pigs and cattle, in the diet is highlighted by the collection of remains during excavation, while the significant role played by the consumption of small fish (<10cm in length) is documented thanks to wet sieving.

The data collected in « La Poubelle des Mamans » illustrate a diet based on fish and livestock products. However, it does not capture the contexts in which these different animals were consumed. In addition, in a context where fish products play an important role, one can only be surprised by the scarcity of scales in the dump. The presence of several large *Polydactylus quadrifilis* (> 50 cm) in the dump gave hope for the discovery of many easily identifiable scales. Does this mean that the fish were scaled elsewhere than where they were cooked? This absence raises questions about the different areas of food preparation and the management of waste from around food preparation areas.
