# Supplementary material for: Investigating grandmothers’ cooking: A multidisciplinary approach to foodways on an archaeological dump in Lower Casamance, Senegal
Source: PLoS One. 2024 May 29;19(5):e0295794. doi: 10.1371/journal.pone.0295794 (PMC11135772; doi:10.1371/journal.pone.0295794)
Supplement: S3 File — (DOCX) [file pone.0295794.s010.docx]

**S3 - Carpology**

**Method**

A total of approximately 96 litres of archaeological soil matrix coming from 8 samples was processed by bucket flotation directly in the field. The ≥0.5 mm sub-samples were screened for charred plant elements such as seeds, fruits, and parenchyma using a low-power stereomicroscope (×10–40). Identification of these elements was made by comparison of archaeological morphotypes with taxonomic specimens in the modern reference collection housed at the Institute of Archaeology, University College London, and isolate samples at the ARCAN laboratory in Geneva. Taxa were identified according to the nomenclature of The Useful Plants of West Tropical Africa [1]. For all the samples analysed, whole vegetal items were counted into a list of taxa, from which relative frequency tables were constructed, and ubiquity calculated. Relative frequency is the percentage of the total seed count. It can be calculated by sample, phase, trench or site, as appropriate. In addition, each taxon was recorded in an ubiquity table. Ubiquity is the number of samples in which a taxon is present as a percentage of the total number of samples (see [2] for more details on the methodology).

**Results**

A total of 37 archaeobotanical items and 184 fragments of carbonised food remains were recovered from 96 L of soil. Three samples of 12 L contain no plant remains. The archaeobotanical results (S3.1 Fig) indicate that the main plant remains from “La Poubelle des Mamans” are domesticated rice (*Oryza Sp.*), which represent about 76% frequency of the assemblage (9 grains, 14 grain fragments and 5 spikelet bases) and is present in two samples (25% ubiquity). Other plant remains are fragments of the endocarp of *Elaeis guineensis* (oil palm tree) (16 fragments for a Minimum Number of Individuals MNI of 6) and 3 caryopses of *Eleusine indica*.

**Fig S3.1: Summary of the archaeobotanical results.** Total numbers, frequency and ubiquity of identified items summarised according to chronological contexts

References

1. Burkill H M. The Useful Plants of West Tropical Africa. Kew: Royal Botanic Gardens; 1985-2000.
2. Champion L, Fuller DQ. Archaeobotany: Methods. In Oxford Research Encyclopedia of African History. 2018.<https://doi.org/10.1093/acrefore/9780190277734.013.204>
