# Supplementary material for: Investigating grandmothers’ cooking: A multidisciplinary approach to foodways on an archaeological dump in Lower Casamance, Senegal
Source: PLoS One. 2024 May 29;19(5):e0295794. doi: 10.1371/journal.pone.0295794 (PMC11135772; doi:10.1371/journal.pone.0295794)
Supplement: S4 File — (DOCX) [file pone.0295794.s011.docx]

**S4 - Morphometrical and use-wear approaches to pottery**

**Method**

The site of “La Poubelle des Mamans” yielded a total of 2746 sherds, 468 pots were individualised and drawn for the morphometric study and 161 of the most complete pots (reconstructed minimum opening diameter, maximum diameter and maximum height), were selected to integrate the study of morphometric groups. All sherds and pots were studied from a use-wear point of view using macroscopic observations (naked eye and stereomicroscope).

While some vessels were sent unwashed for ORA and phytolith analysis, the entire assemblage was washed for the use-wear study phase. Although washing may alter some traces, it is essential to ensure that all traces are visible. In order to limit the washing off of addition traces, such as soot, all sherds were washed by or under the supervision of the use-wear specialist, and all necessary observations were made simultaneously. Use-wear has been identified in two main categories: addition and subtraction of material. The former records the presence or absence of soot deposits, calcareous deposits and organic residues, while the latter records the presence or absence of carbonate dissolution, clay spalling and abrasion marks. This data is put in relation with the position of the wear (internal, external and rim; upper body, medial body, lower body, base, inflexion points) [1].

**Results**

Morphometrical analysis

Each of the reconstructible pots was plotted on a scatterplot combining height and the percentage of aperture ((min opening x 100) / max diameter) (S4.1 Fig) and six morphological groups could be formed. In order of frequency, Group 1, is composed of small to medium sized pots with a restricted or slightly restricted mouth and a twisted cord roulette impression (TCR). Group 2 consists of medium sized pots with an open mouth and are undecorated. Group 3 consists of wide open-mouthed vessels with very different morphologies and functions. Three different sub-groups can be identified: one group displays a prehensile element and can be interpreted as lids according to its shape, a second one consists of pottery in the shape of mortars and a third one consists of pottery wheels. Group 3 did not take part in the integrated functional analysis, although further investigations could be carried out later. Group 4 consists of the largest size vessels in the assemblage with the smallest ratio of mouth diameter to maximum diameter. They are the only vessels decorated all over the body with impressions of rolled shells on the lower part and TCR on the upper part. Group 5 consists of large vessels with a slightly restricted mouth. Lastly, Group 6 consists of large vessels with a wider ratio of rim to maximum diameter than in Group 4, and narrower ratio than in Group 5. The distinction between each group was first established on the basis of morphological criteria and then confirmed by the study of the ornamentation of the pots. This demonstrates that the distinction is already operative when the pot is being made by the potters.

**Fig S4.1: Scatter graph showing the morphological groups of the pottery assemblage according to height and percentage of aperture.**

Use-wear analysis

Macroscopic use-alterations observations show addition traces such as soot deposits, charred residues and lime formation (S4.2), as well as subtractive wear such as abrasions, spalls and dissolution of calcareous temper (S4.3 Fig). This wear and traces are located on the rim and inner or outer walls and are distributed differently on the 161 reconstructible pots. These observations were put in perspective with the morpho-stylistic groups (S4.4 Fig and S4.5 Fig). The correspondence analysis (CA) (S4.3 Fig) shows a strong correlation between the lids of Group 3 and the soot deposits on the inner wall. In order to get a clearer view of the whole assemblage, this group has been removed from figure S4.3 In this new CA plot, we can observe a great similarity between Groups 5 and 1. They show a correlation with limestone accumulation, charred residues, temper dissolution (both heavy and light) and temper dissolution associated with spalls on the internal surface. Group 2 tends to correlate with internal and external spalling. Finally, abrasion located on the rim and inner rim are not precisely correlated with any shape and can be found on any pot, while the absence of traces can be found on any shape but more frequently on Group 4.

**Fig S4.2: Addition traces from the site of La Poubelle des Mamans**

**Fig S4.3: Subtractive wears from the site of La Poubelle des Mamans**

**Fig S4.4: Correspondence Analysis of use-alteration traces according to morpho-stylistic analysis.** a) results with Groups 1, 2, 3 (lids), 4, 5 and 6. Pearson’s Chi squared test = 185.1599, p-value = 5.374969e-16 ; b) results without Group 3 (lids). Pearson’s Chi squared test = 77.88525, p-value = 0.000311663.

**Fig S4.5: Matrigraph of the representation of use-alterations according to morpho-stylistic groups providing detailed quantitative data.**

References

1. Vieugué J. Use-wear analysis of prehistoric pottery: methodological contribution from the study of the earliest ceramic vessels in Bulgaria (6100-5500 BC)”, *Journal of Archaeological Science*, 41, 622–630
