# Supplementary material for: Investigating grandmothers’ cooking: A multidisciplinary approach to foodways on an archaeological dump in Lower Casamance, Senegal
Source: PLoS One. 2024 May 29;19(5):e0295794. doi: 10.1371/journal.pone.0295794 (PMC11135772; doi:10.1371/journal.pone.0295794)
Supplement: S5 File — (DOCX) [file pone.0295794.s012.docx]

**S5 - Organic residue analysis**

**Methods**

Vessels were selected on the basis of morphological and use-wear analyses. Pots showing traces of use over a fire (charred residues and soot) were preferentially sampled on the medial body, as results from previous work demonstrated their relevance [1]. Moreover, pots showing filling limits (indicated by carbonate dissolution), were sampled just below the maximum filling.

The extraction method is based on a commonly used method for lipid extraction from archaeological pottery, slightly modified [2]. The surface of the potsherds was removed with a micro-drill to avoid contamination by exogenous lipids, and they were ground using a mortar and pestle. The extractions were carried out on 3 g of ground pottery spiked with 20 μL of *n*-tetratriacontane (1 mg mL^-1^). 10 mL of a 2:1 dichloromethane/methanol (DCM/MeOH) solution were added to the sample, and the mixture was ultrasonicated for 30 min. After centrifugation, the supernatants were collected and evaporated under a nitrogen flow. Samples were suspended in DCM/MeOH and a fifth was transferred to GC vials. Samples were then derivatised with 50 μL of N,O-bis (trimethylsilyl) trifluoroacetamide (BSTFA) with 1% trimethylchlorosilane (TMCS) and submitted to chromatographic analysis after suspension in cyclohexane.

Chromatographic analyses were first performed on an Agilent 8890 gas chromatograph equipped with a flame ionisation detector (FID) and coupled to an Agilent 5977B mass spectrometer. After injection via an on-column injector, the molecular compounds were separated on a 15 m DB-5HT (Agilent) apolar capillary column with an internal diameter of 0.32 mm and a phase thickness of 0.1 µm, using helium as the carrier gas. The temperature program was as follow: 1 min isothermal at 50 °C followed by a gradient increase to 370 °C at 15 °C min^-1^, followed by 12 min at 370 °C. The output flow was split in two for detection by flame ionisation and mass spectrometry. The mass spectrometer was operated in electron ionisation mode (EI, 70 eV) and mass spectra were acquired over the range *m/z* 50–900.

Samples whose extracts were identified as animal products underwent acid transmethylation to detect additional aquatic molecular markers and to directly measure single compound stable carbon isotopes on palmitic and stearic acid [3].  After the addition of 20 µL of internal standard (n-C_34_), a fresh powder sample was allowed to react with 4 mL MeOH and 800 µL sulphuric acid at 70°C for 4 hours. After centrifugation, the supernatant was extracted 3 times with cyclohexane. The extracts were then evaporated to dryness under a gentle nitrogen flow and suspended in 500 µL cyclohexane. A 100 µL aliquot was derivatised with BSTFA and injected into GC-MS using a SCAN/SIM method designed for the detection of isoprenoid fatty acids and ω-(o-alkylphenyl)alkanoic acids (APAAs). A standard sample, composed of C_16:0_ and C_18:0_ with isotopic values measured by EA-IRMS (LOV laboratory, Villefranche-sur-mer, France), was methylated in the same batch as the archaeological samples to calculate the fractionation caused by methylation.

Injection was carried out in splitless mode, in a Shimadzu GC 2010 PLUS chromatograph coupled to a Shimadzu QP 2010 ULTRA mass spectrometer, fitted with a 60 m DB-23 column (0.25 mm diameter, 0.25 µm film thickness). The temperature rise programme was as follows: 2 min isothermal at 50 °C followed by a gradient increase at 10 °C min^-1^ to 100 °C, at 4 °C min^-1^ to 140 °C, at 0.5 °C min^-1^ to 160 °C, at 20 °C min^-1^ to 250 °C, followed by 10 min at 250 °C. The mass spectrometer was running in electron ionisation mode (EI, 70 eV) and ions *m/z* 74, 87, 213, 270 were recorded for 4,8,12-trimethyltridecanoic acid (TMTD), *m/z* 74, 88, 101, 312 for pristanic acid, *m/z* 74, 101, 171, 326 for phytanic acid and *m/z* 74, 105, 262, 290, 318, 346 for APAAs.

Single compound stable carbon isotopes (C_16:0_ and C_18:0_) measured by GC-C-IRMS (GC Ultra Trace coupled to a Delta V Advantage isotope ratio mass spectrometer) on archaeological samples and the methylation standard were supplied by the CIRAM laboratory (Martillac, France) as part of a service contract.

**Results**

The amount of lipids extracted after DCM/MeOH extraction varies considerably from one vessel to another (5.4 to 260.1 µg g^-1^, S5.1 Table, S5.2 Fig). Palmitic (C_16:0_) and stearic (C_18:0_) acids are common to all samples in varying proportions (C_16:0_/C_18:0_ between 0.47 and 5.43). C_18:1_ is present in most samples, also in varying proportions (C_18:1_/C_18:0_ between 0.05 and 4.00). Oxidation products of C_18:1_, 10-oxo-octadecanoic acid, 10-hydroxyoctadecanoic acid, 9,10-dihydroxyoctadecanoic acid [4] are identified in 5 potsherds. Long-chain fatty acids (LCFA, C_20:0_-C_26:0_), usually associated with plant waxes [5,6], are detected in 6 samples. Branched odd-numbered fatty acids (C_15:0_br, C_17:0_ br) and phytanic acid in 4 samples, and unsaturated odd carbon-numbered fatty acids (C_17:1_) in one sample. These molecules are generally found in ruminant fats and aquatic products, although a bacterial origin cannot be completely excluded for odd-numbered branched fatty acids [7,8]. The percentage of SSR isomer (%SSR) of phytanic acid varies between 65% and 72%, corresponding to the interface between ruminant meat and aquatic resources [8]. The fatty acids are probably largely derived from the partial degradation of triacylglycerols (TAGs), which are present in small amounts in all samples (profiles between T_46_ and T_54_, dominated in most samples by T_48_, and in some by T_50_). Monoacylglycerols (MAG C_16:0_, MAG C_18:1_ and MAG C_18:0_), also degradation products of TAGs, are present in most samples. Polyaromatic hydrocarbons (PAHs) are detected in 7 potsherds. They may have formed within the contents during cooking or may have come from soot deposits [9]. It cannot be excluded that PAHs are related to the firing of the vessels rather than to the cooking of their contents, but their presence only in certain specific vessel forms does not support this hypothesis. Linear alkanes (maximum C_29_) are present in small amounts in 5 vessels and are probably derived from plant waxes. Cholesterol is detected in 2 samples, phytosterols (β-sitosterol, campesterol) in most samples. As they are present in low amounts in all samples, they are probably due to contamination [10] and will not be used for interpretation. 9-octadecenamide and 13-docosenamide are identified in PdM 138.

**Table S5.1: Extraction yields, molecular compositions and stable carbon isotope values in samples studied by ORA.**

**Fig S5.2: Lipid yield and fatty acid ratios in the samples studied in organic residue analysis**

The single compound stable carbon isotopes vary between -30.67‰ and -27.57‰ for C_16:0_ and between -31.21‰ and -27.94‰ for C_18:0_. They correspond to a mixture of animal fats, probably including ruminant carcass fats, porcine fats and/or freshwater (S5.3 Fig). The depletion of ^13^C in one sample (PdM 111) compared to the others suggests that it contains a significant amount of freshwater fat or C_3_ plant.

**Fig. S5.3: Single compound stable carbon isotopes in lipid extracts.** a) δ^13^C_18:0_ values plotted against δ^13^C_16:0_ values. The 95% confidence ellipses are calculated using authentic reference fat values published in the literature [3, 11–24]. b) Δ^13^C values plotted against their δ^13^C_16:0_ value

against δ^13^C_16:0_ values. The 95% confidence ellipses are calculated using authentic reference fat values published in the literature [3, 11–24]. b) Δ^13^C values plotted against their δ^13^C_16:0_ value

**Discussion**

The large amount of lipids and the presence of odd-numbered branched and unsaturated fatty acids and phytanic acid indicate an animal fat content of the PdM 111 vessel (Group 1) and the depleted stable carbon isotope values of C_16:0_ (-30.67‰) and C_18:0_ (-31.21‰) suggest a freshwater origin (S5.3 Fig) [7]. This hypothesis is supported by the co-presence of branched odd-numbered fatty acids, unsaturated odd-numbered C_17:1_, and C_16:0_ and C_18:1_ in high abundance (C_16:0_/C_18:0_=5.4, C_18:1_/C_18:0_=4.0; S5.2 Fig, in turquoise, S5.1 Table) [7,8]. The 72% SSR isomer of phytanic acid in PdM 111 (S5.4 Fig) is not a clear indication of an aquatic source (borderline with values found in ruminant fat [8]), but it correlates well with depleted single compound stable isotopes, compared to other samples with phytanic acid (PdM 3, PdM 49, PdM 51 and PdM 78) which have both lower %SSR (closer to ruminant values) and more enriched ^13^C values (terrestrial range). The absence of ω-(o-alkylphenyl) alkanoic acids (APAAs), markers of thermal transformation of aquatic products, may be due to the fact that the contents of the pot were not heated to a sufficiently high temperature, or for a sufficiently long time, or that the minerals in the paste did not catalyse their formation [28,29]. However, in the absence of other aquatic markers (4,8,12-trimethyltridecanoic and pristanic acids), aquatic products cannot be unambiguously identified, as this combination of molecular and isotopic data could also originate from a mixture of ruminant fat (branched odd-numbered fatty acids, phytanic acid with %SSR isomer not clearly aquatic) and C_3_ plant oil (single compound stable isotopes, high abundance of C_16:0_ and C_18:1_).

**Fig S5.4: Chromatograms of lipid extracts from pot PdM 111**. a) after DCM/MeOH extraction; b) after acid transmethylation (SIM mode analysis). C_xx:x_: fatty acids; C_xx:x_br: branched fatty acids ; TAGs: triacylglycerols; IS: internal standard

PdM 3 (Group 2), PdM 49 (Group 1), PdM 51 and PdM 78 (both Group 5) can be interpreted as cooking vessels, probably for animal products, due to the presence of PAHs and a large amount of lipids (118.0-240.2 µg g^-1^ after extraction with DCM/MeOH, S5.2 Fig, in red), including branched odd-numbered and phytanic acid (S5.2 Fig, S5.1 Table). The %SSR phytanic acid isomer and the proportions of C_16:0_ and C_18:1_ compared to C_18:0_ are lower than in PdM 111 (Group1) (C_16:0_/ C_18:0_=2.4-3.1, C_18:1_/ C_18:0_=0.1-0.6, %SSR=65-69%), suggesting a terrestrial rather than aquatic animal fat [8], but fatty acid ratios must be treated with caution [10]. The presence of 10-oxo-octadecanoic acid, 10-hydroxyoctadecanoic acid, 9,10-dihydroxyoctadecanoic acid indicates the previous occurrence of significant amounts of C_18:1_. The detection of LCFA suggests a plant origin [5,6]. Single compound carbon stable isotopes (-27.97‰ to -27.57‰ for C_16:0_ and -28.55‰ and -27.94‰ for C_18:0_) in these samples suggest a mixed content, composed of ruminant carcass fat and non-ruminant fats (S5.3 Fig). These vessels may be in the same category as PdM 111, with a more complex and degraded signal, possibly related to more regular use and a greater diversity of contents, including animal and plant products.

PdM 6 (Group 6), PdM 48, PdM 72, PdM 90, PdM 94, PdM 127 (all Group 1), PdM 117, PdM 121 (both Group 2) are vessels characterised by low fat content (5.4-32.7 µg g^-1^), sometimes with PAH and LCFA (S5.1 Table). These vessels probably contained low-fat products, possibly of plant origin due to the presence of LCFA, or were used only occasionally. Some of them were probably cooking pots, due to the presence of PAHs.

PdM 71 (Group 5) is characterised by a low lipid content (10.0 µg g^-1^) but shows a high proportion of LCFA (C_20:0_- C_26:0_, S5.1 Table), which is typical of plant waxes [5,6]. The very high proportion of C_16:0_ and C_18:1_ (C_16:0_/C_18:0_=3.7, C_18:1_/C_18:0_=3.8, S5.2 Fig, in green) also support the plant hypothesis. The low-lipid plant product in this pot suggest that it may have been used as a boiling vessel for medicinal plants, a hypothesis also mentioned to interpret the presence of plant wax in Nok pottery from Nigeria [5].

The contents of PdM 120 (Group 2) and PdM 138 (Group 4) are more difficult to interpret (S5.2 Fig, in dark blue and purple, S5.1 Table). The moderate amounts of lipid (23.6 µg g^-1^), the very high proportion of C_16:0_ to C_18:0_ (C_16:0_/C_18:0_=4.6, C_18:1_/C_18:0_=0.9) in PdM 120 suggest a product of plant or aquatic origin. In the case of PdM 138, the low extraction yield after direct acid methylation (18.2 µg g^-1^) suggests that the relatively high yield after extraction with DCM/MeOH (63.3 µg g^-1^) is due to contamination. The presence of 9-octadecenamide and 13-docosenamide in this extract supports this interpretation, as they could originate from plastic material [25], or from microorganisms (these molecules are synthesised by fungi of the genus Aspergillus which grow in the soil and on food) [26, 27].

References

1. Drieu L, Regert M, Mazuy A, Vieugué J, Bocoum H, Mayor A. Relationships Between Lipid Profiles and Use of Ethnographic Pottery: an Exploratory Study. J Archaeol Method Theory. 2022; 29: 1294–1322. <https://doi.org/10.1007/s10816-021-09547-1>
2. Evershed RP, Heron C, Goad LJ. Analysis of organic residues of archaeological origin by high-temperature gas chromatography and gas chromatography-mass spectrometry. Analyst. 1990; 115: 1339–1342. <https://doi.org/10.1039/AN9901501339>
3. Craig OE, Saul H, Lucquin A, Nishida Y, Taché K, Clarke L, Thompson A, Altoft DT, Uchiyama J, Ajimoto M, Gibbs K, Isaksson S, Heron CP, Jordan P. Earliest evidence for the use of pottery. Nature. 2013; 496: 351–354. Https://doi.org/10.1038/nature12109
4. Berstan R, Dudd SN, Copley MS, Morgan ED, Quyec A, Evershed RP. Characterisation of “bog butter” using a combination of molecular and isotopic techniques. Analyst. 2004; 129: 270–275. <https://doi.org/10.1039/b313436a>
5. Dunne J, Höhn A, Neumann K, Franke G, Breunig P, Champion L, et al. Making the invisible visible: tracing the origins of plants in West African cuisine through archaeobotanical and organic residue analysis. Archaeol Anthropol Sci. 2022; 14: 30. <https://doi.org/10.1007/s12520-021-01476-0>
6. Dunne J, Mercuri AM, Evershed RP, Bruni S, di Lernia S. Earliest direct evidence of plant processing in prehistoric Saharan pottery. Nature Plants. 2016; 3: 16194. [https://doi.org/](https://doi.org/10.1039/AN9901501339)[10.1038/nplants.2016.194](https://doi.org/10.1038/nplants.2016.194)
7. Cramp L, Evershed RP. Reconstructing aquatic resource exploitation in human prehistory using lipid biomarkers and stable isotopes. In: Holland H, Turekian KK, editors. Treatise on Geochemistry: Archaeology and Anthropology. Amsterdam: Elsevier; 2014. pp. 319–339.
8. Lucquin A, Colonese AC, Farrell TF, Craig OE. Utilising phytanic acid diastereomers for the characterisation of archaeological lipid residues in pottery samples. Tetrahedron Letters. 2016; 57: 703–707. [https://doi.org/](https://doi.org/10.1038/s41598-020-78332-z)[10.1016/j.tetlet.2016.01.011](https://doi.org/10.1016/j.tetlet.2016.01.011)
9. Craig OE. Organic analysis of «food crusts» from sites in the Schelde valley, Belgium: a preliminary evaluation. Notae Praehistoricae. 2004; 24: 209–217.
10. Whelton HL, Hammann S, Cramp LJE, Dunne J, Roffet-Salque M, Evershed RP. A call for caution in the analysis of lipids and other small biomolecules from archaeological contexts. Journal of Archaeological Science. 2021;132: 105397. <https://doi.org/10.1016/j.jas.2021.105397>
11. Bondetti M, González Carretero L, Dolbunova E, McGrath K, Presslee S, Lucquin A, et al. Neolithic farmers or Neolithic foragers? Organic residue analysis of early pottery from Rakushechny Yar on the Lower Don (Russia). Archaeol Anthropol Sci. 2021;13: 141. doi:10.1007/s12520-021-01412-2
12. Choy K, Potter BA, McKinney HJ, Reuther JD, Wang SW, Wooller MJ. Chemical profiling of ancient hearths reveals recurrent salmon use in Ice Age Beringia. Proceedings of the National Academy of Sciences. 2016;113: 9757–9762.
13. Courel B, Robson HK, Lucquin A, Dolbunova E, Oras E, Adamczak K, et al. Organic residue analysis shows sub-regional patterns in the use of pottery by Northern European hunter–gatherers. Royal Society Open Science. 2020;7: 192016. doi:10.1098/rsos.192016
14. Craig OE, Steele VJ, Fischer A, Hartz S, Andersen SH, Donohoe P, et al. Ancient lipids reveal continuity in culinary practices across the transition to agriculture in Northern Europe. Proc Natl Acad Sci. 2011;108: 17910–17915. doi:10.1073/pnas.1107202108
15. Cramp LJ, Jones J, Sheridan A, Smyth J, Whelton H, Mulville J, et al. Immediate replacement of fishing with dairying by the earliest farmers of the northeast Atlantic archipelagos. Proceedings of the Royal Society B: Biological Sciences. 2014;281: 20132372. doi:10.1098/rspb.2013.2372
16. Debono Spiteri C. Pottery use at the transition to agriculture in the western Mediterranean. Evidence from biomolecular and isotopic characterisation of organic residues in Impressed/Cardial Ware vessels. PhD, University of York. 2012.
17. Drieu L, Lucquin A, Cassard L, Sorin S, Craig OE, Binder D, et al. A Neolithic without dairy? Chemical evidence from the content of ceramics from the Pendimoun rock-shelter (Castellar, France, 5750-5150 BCE). Journal of Archaeological Science: Reports. 2021;35: 102682. doi:10.1016/j.jasrep.2020.102682
18. Dudd SN. Molecular and isotopic characterisation of animal fats in archaeological pottery. PhD, University of Bristol. 1999.
19. Gregg MW. Organic residue analysis and the earliest uses of pottery in the ancient Middle East. PhD, University of Toronto. 2009.
20. Horiuchi A, Miyata Y, Kamijo N, Cramp L, Evershed RP. A dietary study of the Kamegaoka culture population during the Final Jomon Period, Japan, using stable isotope and lipid analyses of ceramic residues. Radiocarbon. 2015;57: 721–736.
21. Lucquin A, Gibbs K, Uchiyama J, Saul H, Ajimoto M, Eley Y, et al. Ancient lipids document continuity in the use of early hunter–gatherer pottery through 9,000 years of Japanese prehistory. Proceedings of the National Academy of Sciences. 2016;113: 3991–3996. doi:10.1073/pnas.1522908113
22. Outram AK, Stear NA, Bendrey R, Olsen S, Kasparov A, Zaibert V, et al. The Earliest Horse Harnessing and Milking. Science. 2009;323: 1332–1335. doi:10.1126/science.1168594
23. Pääkkönen M, Evershed RP, Asplund H. Compound-specific stable carbon isotope values of fatty acids in modern aquatic and terrestrial animals from the Baltic Sea and Finland as an aid to interpretations of the origins of organic residues preserved in archaeological pottery. Journal of Nordic Archaeological Science. 2020;19.
24. Taché K, Craig OE. Cooperative harvesting of aquatic resources and the beginning of pottery production in north-eastern North America. Antiquity. 2015;89: 177–190.
25. Barnard H, Dooley A, Faull K. An introduction to archaeological lipid analysis by combined gas chromatography mass spectrometry (GC/MS). In: Barnard H, Eerkens JW, editors. Theory and practice of archaeological residue analysis. Oxford: Archaeopress; 2007. pp. 42–60.
26. Giannoukos K, Giannoukos S, Lagogianni C, Tsitsigiannis DI, Taylor S. Analysis of volatile emissions from grape berries infected with Aspergillus carbonarius using hyphenated and portable mass spectrometry. Sci Rep. 2020; 10: 21179. <https://doi.org/10.1038/s41598-020-78332-z>
27. Pennerman KK, AL-Maliki HS, Lee S, Bennett JW. Fungal Volatile Organic Compounds (VOCs) and the Genus Aspergillus. New and Future Developments in Microbial Biotechnology and Bioengineering. Elsevier; 2016. pp. 95–115. <https://doi.org/10.1016/B978-0-444-63505-1.00007-5>
28. Copley MS, Hansel FA, Sadr K, Evershed RP. Organic residue evidence for the processing of marine animal products in pottery vessels from the pre-colonial archaeological site of Kasteelberg D east, South Africa. South African Journal of Science. 2004;100: 279–283.
29. Bondetti M, Scott E, Courel B, Lucquin A, Shoda S, Lundy J, et al. Investigating the formation and diagnostic value of ω-(o-alkylphenyl)alkanoic acids in ancient pottery. Archaeometry. 2021;63: 594–608. doi:10.1111/arcm.12631
