# Supplementary material for: Investigating grandmothers’ cooking: A multidisciplinary approach to foodways on an archaeological dump in Lower Casamance, Senegal
Source: PLoS One. 2024 May 29;19(5):e0295794. doi: 10.1371/journal.pone.0295794 (PMC11135772; doi:10.1371/journal.pone.0295794)
Supplement: S6 File — (DOCX) [file pone.0295794.s013.docx]

**S6 - Microbotanical remains**

**Method**

Phytoliths are microscopic opal particles that precipitate within cells and/or between living plant tissues. The identification of phytoliths in residues of pottery represents a new opportunity to provide direct evidence of cooking practices and to identify dietary patterns. Firstly, because of their siliceous structure, phytoliths are more resistant to destruction by heat and extreme pHs than other biological proxies [1,2,3]. Secondly, the possibility to differentiate the parts of plants (seeds, leaves, roots, bark), Poaceae subfamilies and charred phytoliths can provide detailed information about the contents and the functions of pottery. Fifteen ceramics plots were examined for this study. These ceramics were chosen to represent the diversity of the different groups identified by morphometric and use-wear analysis. Phytolith sampling was carried out by scraping on the inner part of the pottery walls. We assume that phytolith assemblages may originate from the plant residues adhering to the inside of the pots but also from the ceramic paste. Samples were prepared at the LGP-CNRS (Paris) according to Piperno (2006) and studied as followed for sediment samples : (1) deflocculation of sediments in a solution of sodium hexametaphosphate (Na_2_CO_3_); (2) sieving at 250 μm mesh ; (3) clay removal by decantation; (4) destruction of organic matter with a solution of H_2_O_2_ at 130%; (5) phytoliths extraction by densimetric separation with a heavy liquid (sodium polytungstate Na_6_) set at d = 2.30–2.35; and (6), mounting of samples on microscope slides using oil immersion to allow the observation of phytoliths in three dimensions. The classification of phytolith morphotypes is based on the ICPN 2.0 classification [4] and information from the literature.

**Results and discussion**

The fifteen samples analysed are relatively rich in phytoliths and show fairly similar assemblages with a strong dominance of phytoliths produced by Arecaceae (S6.1 Fig). Indeed, their values reach between 32 and 65% of the assemblages. Phytoliths produced by woody and herbaceous dicotyledons are also well represented with values between 10 and 29% depending on the sample.  We also note in several assemblages the presence of morphotypes produced by Cyperaceae or Commelinaceae herbs. Like the high proportion of tree and palm phytoliths, the presence of these adventice morphotypes calls into question the origin and interpretation of these phytoliths, which seem to originate more from the surrounding vegetation of the “Poubelle des mamans”. However, the interpretation of the phytoliths produced by the palm trees is complex. If they can represent pollution from the surrounding palm trees they are also widely used in local recipes (palm oil, palm wine...).

**Fig S6.1: Phytolith diagram of the 15 pots of La Poubelle des Mamans**

The percentage phytolith diagram has been organised according to the different groups of pots identified by morphometric and use-wear analysis (S6.1 Fig)

For group 1, the presence of the diagnostic morphotype Papillate in the assemblages of three pots (PdM 48, PdM 127, PdM 90) suggests the use of grass inflorescences such as grains (ICPN, 2019). This is also evidenced for the PdM 90 by the high amount of elongate dendritic (7%), which are commonly formed in the epidermis of inflorescence bracts such as glume (ICPN, 2019). Poaceae are also well identified in the PdM 72 by the Grass Silica Short cell Phytoliths (GSSCP) reaching 15,7% while they represent only 2,9% for the PdM 123. Moreover, the low abundance of phytoliths in this sample suggests a non-vegetable origin.

The phytolith signal of the four pots from the group 2 (PdM 3, 117, 120 and 121) is unclear and doesn't really allow a vegetal interpretation. All phytolith groups are observed without clear diagnostic morphotypes or association of morphotypes.

The interpretation of PdM 138 (group 4) is facilitated by the presence of both the Scooped bilobate GSSCP produced by the Ehrhartoideae subfamily, to which rice belongs, and the Papillate. It suggests the use of grass seeds, certainly domestic rice (Oryza sp.). In contrast, no phytolith signal was observed for the PdM 6.

The phytolith assemblages of pots from the group 5 (PdM 51, 71, 78) are not easy to interpret. Two interesting morphotypes are observed in two pots: scooped bilobate GSSCP in the PdM 78 and Papillate in the PdM 51 suggesting the use of grasses, probably rice, in these two pots. However, the phytolith signal is weak due to the high contamination of the surrounding vegetation and the phytoliths contained in the ceramic paste.

References

1. Piperno DR. Phytoliths: A Comprehensive Guide for Archaeologists and Paleoecologists. Lanham, MD: AltaMira Press; 2006.
2. Saul H, Madella M, Fischer A, Glykou A, Hartz S, Craig OE. Phytoliths in pottery reveal the use of spice in European prehistoric cuisine. PloS one. 2013;8: e70583.
3. 3. Hart, John P, Robert G. Thompson, and Hetty Jo Brumbach. (2003). Phytolith Evidence for Early Maize (Zea Mays) in 2003 the Northern Finger Lakes Region of New York. American Antiquity 68(4), 619-640.
4. 4. International Committee for Phytolith Taxonomy (ICPT) Neumann, K., Strömberg C.A.E; Ball, T., Albert, R.M., Vrydaghs, L., Scott-Cummings L. (2019). International code for phytolith nomenclature (ICPN) 2.0. Annals of Botany, 124(2), 189-199.
